# Nanomechanical detection to empower robust monitoring of sepsis and microbial adaptive immune system-mediated proinflammatory disease

**DOI:** 10.1038/s41598-024-80126-6

**Published:** 2024-12-02

**Authors:** Kessarin Thanapirom, Walid Al-Akkad, Aylin Pelut, Zahra Sadouki, Jemima B. Finkel, Stefan Nardi-Hiebl, Wieland Vogt, Benjamin Vojnar, Hinnerk Wulf, Leopold Eberhart, Timothy D McHugh, Krista Rombouts, Massimo Pinzani, Emmanouil Tsochatzis, Joseph W. Ndieyira

**Affiliations:** 1 Division of Medicine, University College London, Gower Street, London, WC1E 6BT United Kingdom; 2 UCL Centre for Clinical Microbiology, Division of Infection and Immunity, University College London, Gower Street, WC1E 6BT London, United Kingdom; 3 Department of Anaesthesia and Intensive Care, University Hospital of the Philipps-University of Marburg Baldingerstrasse, 35043 Marburg, Germany; 4 Medical Innovations and Management, Steinbeis University, Ernst-August-Strasse 15, 12489 Berlin, Germany

**Keywords:** Biophysics, Health care, Nanoscience and technology

## Abstract

The correlation between circulating microbes and sepsis as well as proinflammatory diseases is increasingly gaining recognition. However, the detection of microbes’ cell-free DNA (cfDNA), which exist at concentrations of a billion times lower than blood proteins, poses a significant challenge for early disease detection. Here, we present Nano mechanics combined with highly sensitive readout sequences to address the challenges of ultralow counts of disease biomarkers, thus enabling robust quantitative monitoring of chronic medical conditions at different stages of human disease progression. To showcase the effectiveness of our approach, we employ fragments of cfDNA and human cell secretory proteins as models with predictive capabilities for human diseases. Notably, our method reveals a reliable representation over an impressive three to four orders of magnitude in the detection limit and dynamic range, surpassing commercially available quantitative polymerase chain reaction (qPCR) commonly used in routine clinical practice. This concept underpins a highly sensitive and selective medical device designed for the early detection of circulating microbes in patients undergoing intensive cancer therapy. This will help pinpoint individuals at risk of complications, including damage to the intestinal barrier and development of neutropenic fever/Sirsa/Sepsis. Moreover, this approach introduces new avenues for stratifying antibiotic prophylaxis in proinflammatory diseases.

**Supplementary Information:**

The online version contains supplementary material available at 10.1038/s41598-024-80126-6.

## Introduction

Proinflammatory-mediated diseases, such as Crohn’s disease, liver cancer, rheumatoid arthritis, multiple sclerosis, cardiovascular disease (CVD), and diabetes, impact millions of people worldwide. These diseases display diversity in clinical and immunological aspects, with emerging evidence highlighting the significant role of gut microbiota in their development^1–4^. Diabetes and CVD are major risk factors for hospitalisation and mortality, especially in COVID-19 infections^5^. Humans and animals host diverse microbial ecosystems, impacting health-related processes. The gut, skin, and oral cavity serve as ecological niches and sources of pathogens in human blood circulation^6^. Dysbiosis, an imbalance in a person’s natural microflora, enables the gut microbiome to enter the circulatory system due to compromised epithelial barriers. Gut microbial dysbiosis significantly contributes to the pathophysiology of gastrointestinal diseases, including Crohn’s disease^7^, non-alcoholic steatohepatitis (NASH)^8^, and advanced liver cirrhosis^9^. Despite immune-mediated proinflammatory molecules targeting to restore tissue homeostasis, chronic conditions like rheumatoid arthritis, multiple sclerosis, and liver fibrosis orchestrate persistent inflammation, resulting in sustained damage. These conditions often arise from recurrent insults by bacterial infections or toxins, triggering proinflammatory mediators, perpetuating inflammation, and causing tissue dysfunction and failure.

Blood serves as a rich source of information, providing reliable biomarkers crucial for early disease detection. Among these biomarkers, cfDNA stands out due to its well-suited structural motif^10^. It serves as a critical biomarker for studying prevalent human diseases and supports therapeutic applications like gene therapy^11^. Studies have shown the connection between chronic bacterial infections, cfDNA, and their pivotal role in cancer-promoting features and immune-mediated proinflammatory diseases^12,13^. Additionally, bacteria-wide association studies recognise cfDNA as an essential disease indicator, offering potential guidance for clinical interventions. The integration of microbial cfDNA with other diagnostic biomarkers in a diagnostic platform holds the promise of rapid and accurate identification of suspected diseases^10^. This approach would prompt early and accurate identification of suspected diseases, thus facilitating correct treatment and eliminate ineffective antibiotic prescriptions.

Detecting microbial cfDNA and secretory proteins usually requires costly and time-consuming diagnostic technologies that rely on centralised lab equipment. Conventional methods face difficulty in delivering precise and reproducible quantitative assessments with high specificity and sensitivity, particularly for cfDNA and secretory proteins in blood circulation, which have concentrations orders of magnitude lower than blood serum proteins. Currently, various methods are used for measuring microbial cfDNA signatures, including competent culture enrichment techniques like blood agar. However, these methods have limitations, such as long analysis times (> 30 hrs) before results are known. While qPCR is increasingly adopted to simplify the process and enhance cfDNA target yield, its high reagent costs and logistical demands in multistep sample preparations hinder widespread adoption^14^. The CRISPR-Cas-based genomic editing technique, endorsed by Ackerman and co-workers, offers massively multiplexed nucleic acid detection but comes with challenges such as ‘on- or off-target’ modifications compromising accuracy and resolution^15,16^. Genome sequencing, another extensively used technique, demands meticulous sample preparations, and its outcome is greatly influenced by the quality and quantity of DNA or RNA extracted from samples^17^. Additionally, such meticulous sample preparations may introduce biases or artifacts that can impact on downstream analyses. To overcome these barriers, there is a pressing need for the development of high-level multiplexing, more cost-effective, and user-friendly diagnostic technologies. These advancements should enable simultaneous detection of multiple biomarkers in a single patient sample, fostering broader adoption of biomarker panels in clinical settings and facilitating more effective point-of-care screening for inflammatory and cancer-related diseases.

Here, we present a novel multiplexed nanomechanical approach capable of generating measurable and consistent signals for features associated with inflammatory and cancer-related diseases. This technology has previously been used to assess cardiac contractility^18^and perform bacterial susceptibility assays^19,20^. Additionally, it has been employed to analyse biochemical interactions for the detection of cancer biomarkers^21–23^and vaccine characterisation^24^, with unprecedented sensitivity. Our interest in this technology began with pioneering work on nanomechanical sensors, which provided valuable insights into antibiotic binding to mucopeptides and the mechanisms driving superbug drug resistance^25^. Building on this foundation, we developed surface-stress sensors capable of rapid, ultrasensitive detection of active free drugs in human serum, marking a significant advancement in diagnostic capabilities^26^. Further innovations allowed us to decouple surface binding kinetics and reconfigure receptor footprints, enhancing the sensitivity of stress assays^27^. Our exploration of surface-mediated cooperative interactions revealed that these interactions can amplify mechanical forces, thereby increasing the efficacy of antibiotics^28^. More recently, we have enhanced this technology to improve the sensitivity and reproducibility of nanomechanical sensing in living cells, paving the way for real-time cellular analysis^29^. Furthermore, our integration of optical diffraction techniques for alignment-free, precise measurements of nanomechanical bending has expanded the capabilities of this field^30^. While these advancements highlight the critical role of nanomechanical sensing in advancing biomedical research, its potential application in assessing in-vitro models for maintaining host–microorganism homeostasis or evaluating intestinal barrier damage caused by aggressive chemotherapy remains unexplored.

As part of our novel approach to advancing this technology for more effective point-of-care screening of inflammatory and cancer-related diseases, we systematically verified the feasibility of detecting microbial cfDNA signatures and secretory proteins simultaneously on a single biosensor chip. We hypothesise that nanomechanical assays can be tailored to produce distinct signals, even in the presence of high biological noise from interfering agents such as blood serum, plasma components, and unfiltered cell media supernatant. This is possible because each nanomechanical sensor on a single chip function independently, and the specificity of molecular recognition is determined by the complementarity between antigens or ligands in solution and the receptor or capture molecules immobilized on the surface.

**Fig. 1:**
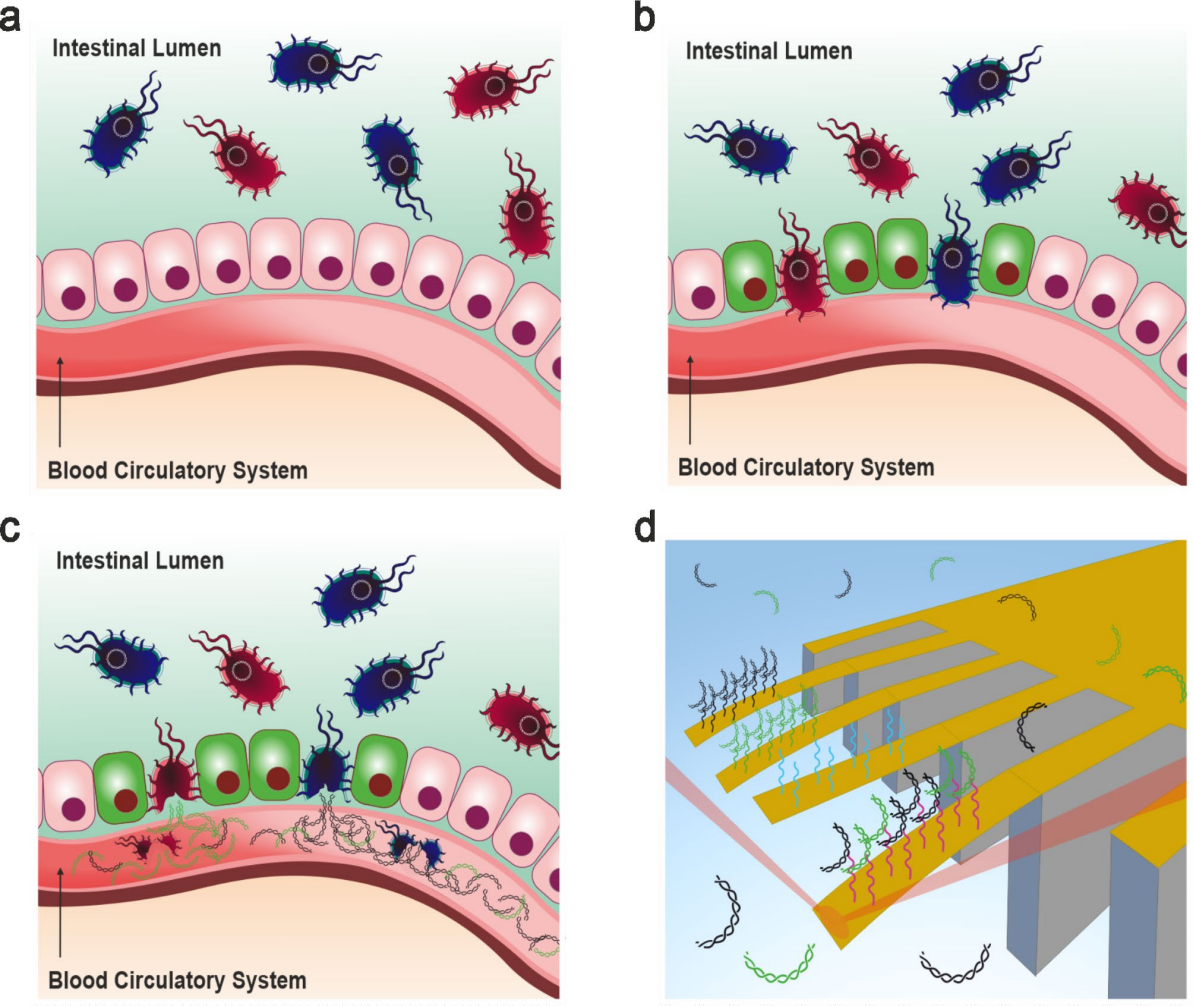
Ultrasensitive detection of markers for proinflammatory mediated diseases. (**a**) Schematic diagram showing a model of blood circultory system (red cavity) in a healthy gut with steady state homeostasis established by a microbiome richness and diversity including Gram-positive (red catoon) and Gram-negative (blue) bacterial species. (**b**) Schematic diagram showing a damaged intestinal barrier in which bacteria accumulate and exert pressure against the epithelial layer. (**c**) The corresponding model in which a damaged intestinal epithelial-barrier increases the permeability of bacteria into the circultory system. The bacteria released DNA is then fragmented by intrinsic factors, that leads to a rise of bacterial cfDNA in the circulatory system. (**d**) Illustration of basic nanomechanical detection system consists of three major units; (1) mechanical elements (yellow), each 500 μm long, 100 μm wide and 1 μm thick, (2) sensing module including capture agents represented by vertical sticks; universal capture (magenta), Gram-negative capture (black), Gram-positive sequences (green) and negative control (light blue) and (3) sample dispensing system used to deliver sample is highlighted by the light blue shed and has a total volume of 80 µl. The recognition step sensing sequences and ligands delivered in serum plasma causes the mechanical elements to deflect vertically and it is monitored in parallel using time-multiplexed optical beam (red) detection on a single photodetector. The results of blood microbiome detection are particularly advantageous for the differential diagnosis in various clinical settings, including in patients after aggressive chemotherapy regimens, which can lead to the damage of the intestinal barrier. These tests may be used as an indicator of the extend of intestinal barrier damage and will help identify patients at risk of neutropenic fever/SIRS/Sepsis as well as for providing valuable information to stratify the antibiotic prophylaxis.

We tested this hypothesis using a range of model biomarkers, including microbial cfDNA signatures, human serum albumin, human DNA, CA19-9 antigens, and Chromogranin A (CgA), the latter of which is commonly used in clinical settings to differentiate between pancreatic ductal adenocarcinoma (PDAC) and pancreatic neuroendocrine tumours (PNET). Our initial focus was on bacterial antigens, which are known to trigger a persistent proinflammatory state, a precursor to immune system-mediated proinflammatory diseases and hepatocellular carcinoma^8^. As shown in Fig. 1, we evaluated the specificity and sensitivity of quantitative monitoring for cfDNA and secretory proteins as expected at different stages of human disease progression, without requiring multistep sample extraction, amplification, or labeling. High-resolution cfDNA detection was achieved by utilizing differential competing stresses on the top and bottom surfaces of a nanomechanical cantilever biosensor, which results in vertical deflection of the sensing element.

**Fig. 2:**
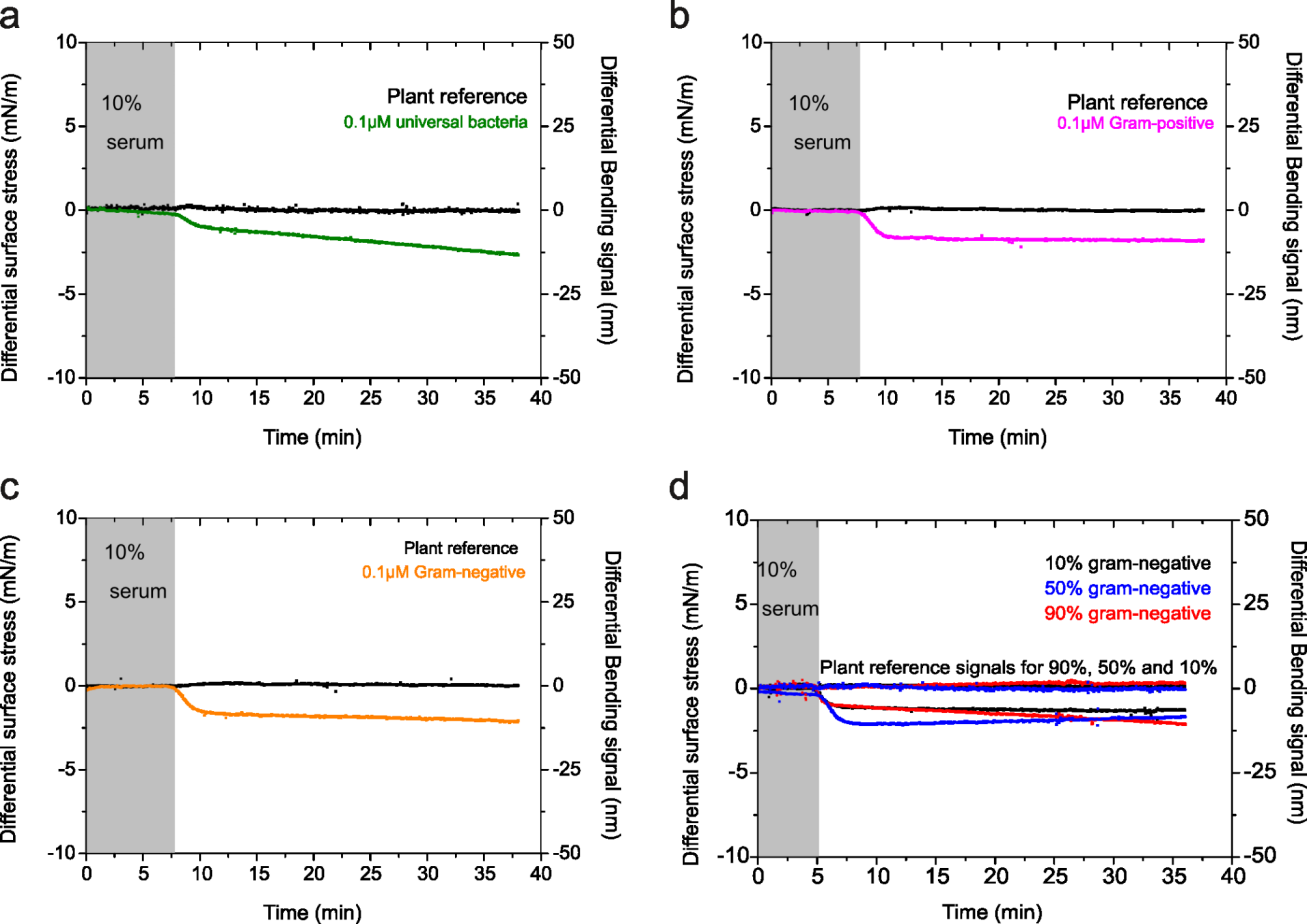
Detection of Gram-negative and Gram-positive bacteria. (**a**) The differential surface stress-versus-time data recorded for bacterial sequence surface construct after delivery of 0.1 µM model bacterial DNA of 27 bp fragment length (green curve). (**b**) The differential surface stress-versus-time data recorded for bacterial sequence surface construct after delivery of 0.1 µM model Gram-positive bacterial DNA of 28 bp fragment length (magenta curve). (**c**) The differential surface stress-versus-time data recorded for bacterial sequence surface construct after delivery of 0.1 µM model Gram-negative bacterial DNA of 28 bp fragment length (orange curve). (**d**) Change in differential surface stress versus concentration data recorded after delivery of 0.1 µM model Gram-negative bacterial DNA fragments in solution in the percentage ratios of 90% (red), 50% (blue) and 10% (black) to 0.1 µM model Gram-positive bacterial DNA fragments. Here all three curves, within experimental error, are consistent in indicating universality of bacterial detection. In (**a**-**d)**, The differential plant reference signal is shown in black, the differential measurements were acquired in a complex liquid media at 30 µl/min; 10% human blood serum plasma diluted in 1 M NaCl sodium citrate buffer (SCC), pH = 7.4 and the grey shaded area represents the injection of serum solution without the bacterial DNA for control measurements (lasting for 5–7 min), to establish a baseline. The results show that direct mechanical assays offer the potential to reliably identify bacterial signatures from human blood serum sample under 5 min.

**Fig. 3:**
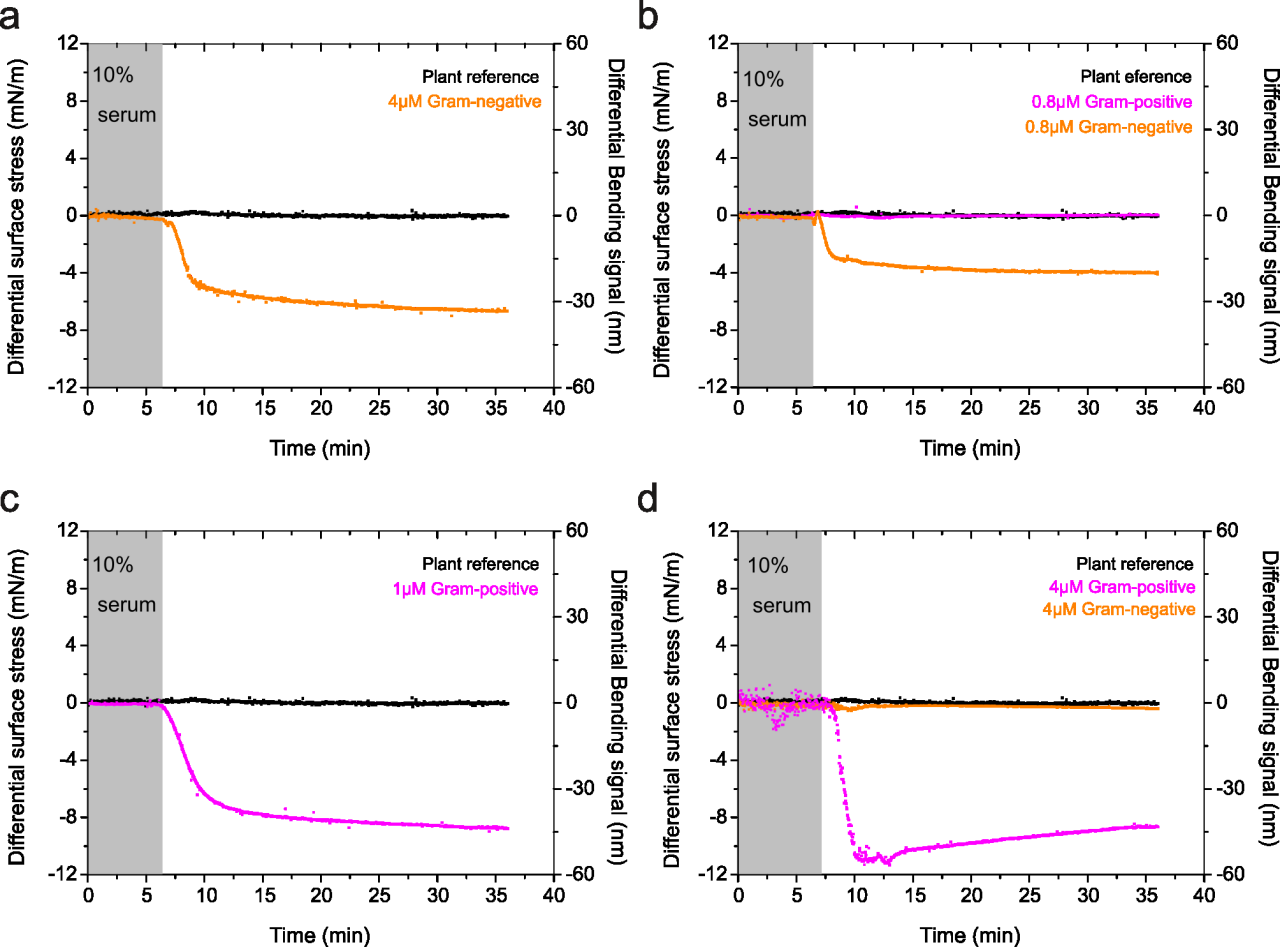
Multiplexed detection of disease markers using direct mechanical assays. (**a**) The differential surface stress-versus-time data recorded for Gram-negative sequence surface construct after delivery of 4 µM model Gram-negative bacterial DNA (orange curve). (**b**) The differential surface stress-versus-time data recorded simultaneously from two sensing mechanical elements in an array, where they were functionalized with Gram-negative sequences. The mechanical response corresponds to times when the solutions of 0.8 µM Gram-negative DNA (orange curve) and 0.8 µM Gram-positive DNA (magenta curve) were simultaneously delivered to the mechanical sensors. (**c**) The differential surface stress-versus-time data recorded for Gram-positive sequence surface construct after delivery of 1 µM model Gram-positive bacterial DNA (magenta curve). (**d**) The differential surface stress-versus-time data recorded simultaneously from two sensing mechanical elements in an array, where they were functionalized with Gram-positive sequences. The mechanical response corresponds to times when the solutions of 4 µM Gram-positive DNA (magenta curve) and 4 µM Gram-negative DNA (orange curve) were simultaneously delivered to the mechanical sensors. In (**a**-**d)**, The differential plant reference signal is shown in black, the differential measurements were acquired in a complex liquid media at 30 µl/min; 10% human blood serum plasma diluted in 1 M NaCl sodium citrate buffer (SCC), pH = 7.4 and the grey shaded area represents the injection of serum solution without the bacterial DNA for control measurements (lasting for 6 min), to establish a baseline. The results show multiplexed detection of multiple disease markers with high selectivity and sensitivity and can be used to distinguish unambiguously the bacterial signatures in the blood serum plasma, such as quantity and identity.

**Fig. 4:**
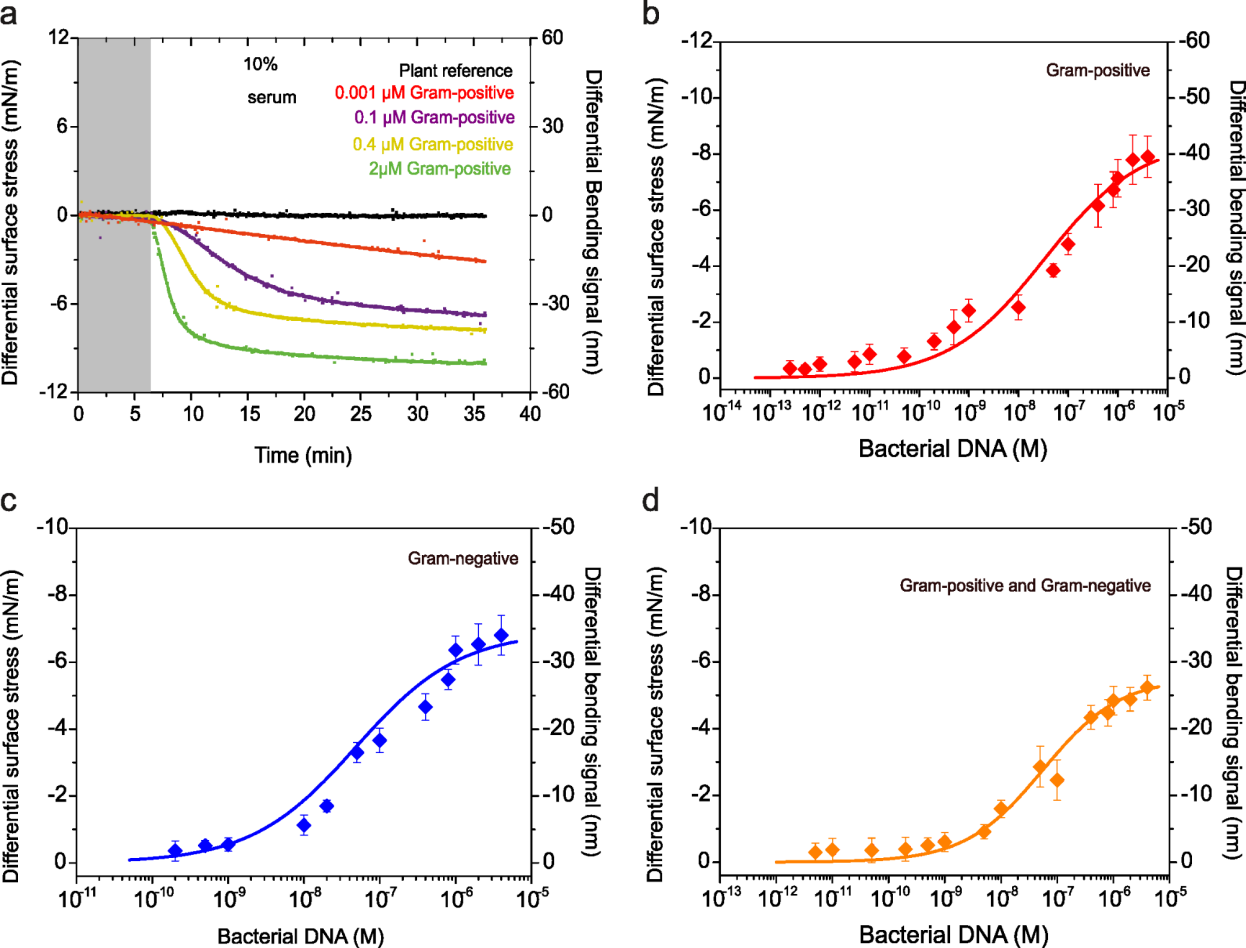
Quantitation of time-resolved selectivity and sensitivity. (**a**) The differential surface stress-versus-concentration of Gram-positive DNA for Gram-positive sequence surface construct. The delivery of Gram-positive bacterial DNA concentrations were 0.001 µM (red curve), 0.1 µM (purple curve), 0.4 µM (yellow curve) and 2 µM (green curve), respectively. The differential plant reference signal is shown in black, the differential measurements were acquired in a complex liquid media at 30 µl/min; 10% human blood serum plasma diluted in 1 M NaCl sodium citrate buffer (SCC), pH = 7.4 and the grey shaded area represents the injection of serum solution without the bacterial DNA for control measurements (lasting for 6 min), to establish a baseline. (**b**) The corresponding semi-log plot showing the differential surface stress-versus-concentration of Gram-positive DNA (experimental data, with estimated standard deviation error bars are shown as solid diamond symbols and lines in red) as a function of concentrations in solution. (**c**) A semi-log plot showing the differential surface stress-versus-concentration of Gram-negative DNA (experimental data, with estimated standard deviation error bars are shown as solid diamond symbols and lines in blue) as a function of concentrations in solution against Gram-negative sequences. (**d**) A semi-log plot showing the differential surface stress-versus-concentration of bacterial DNA (experimental data, with estimated standard deviation error bars are shown as solid diamond symbols and lines in orange) as a function of concentrations in solution against bacterial sequences. In (**b**-**d)**, The three curves superimposed on the data correspond to equilibrium differential surface stress values calculated in the presence of Gram-positive species, Gram-negative species and model bacteria according to Eq. (1), using the following estimates of the maximum surface stress at full coverage σ_*max*_, surface equilibrium dissociation constant *K*_*d*_ and *n* the stoichiometric coefficient. The surface stress data error bars were determined as the standard deviation of the surface stress data fitted measurements from three separate nanomechanical chips. The results confirms selective and high-sensitivity detection of bacterial signatures in a simple and yet robust detection system from a complex biological background.

**Fig. 5:**
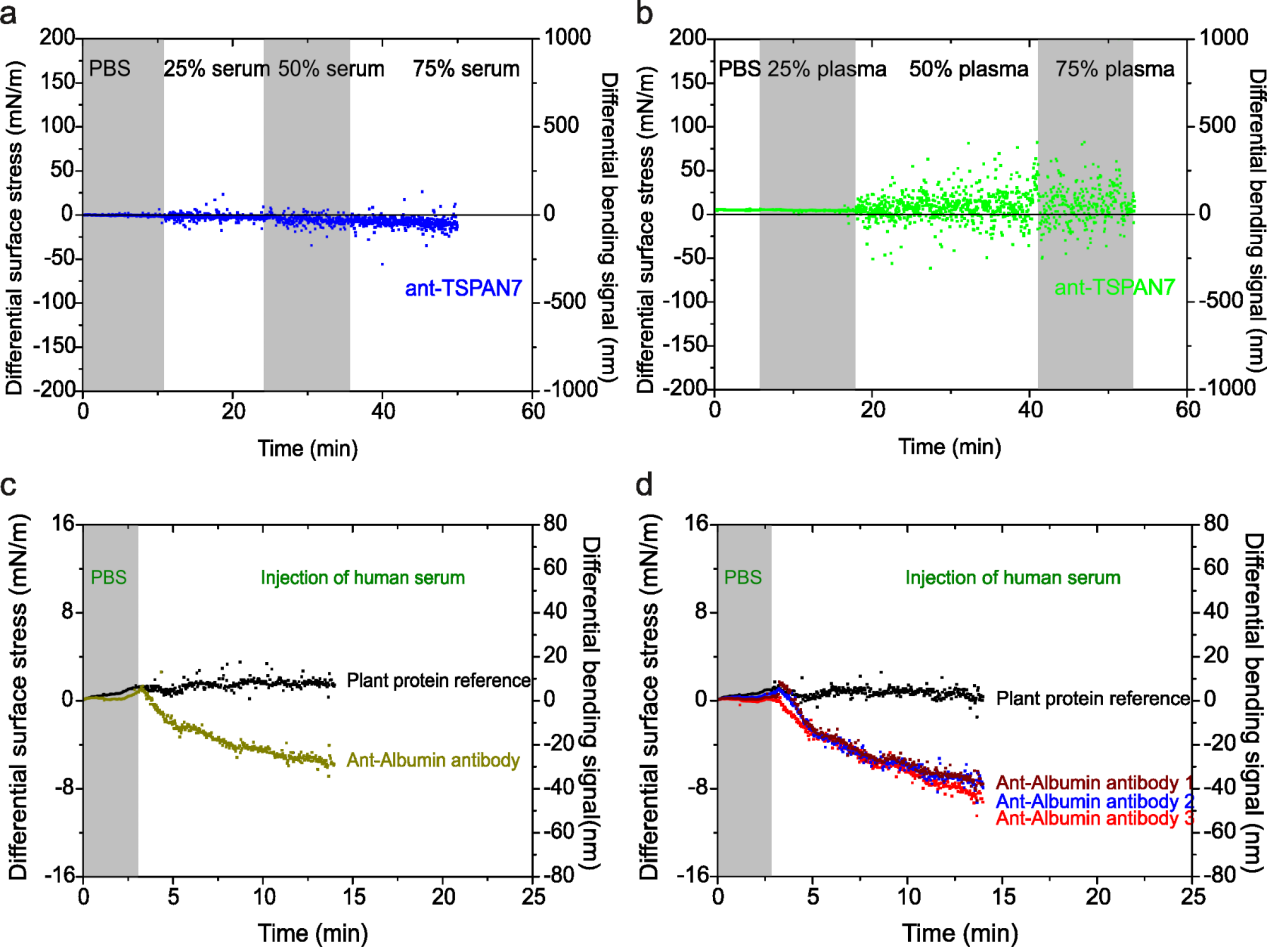
Investigating the biological noise and specificity of biomarker detection. (**a**) Differential deflection of 50 M anti-TSPAN7 monoclonal antibody coated cantilever (blue) upon injection of diluted serum solution concentrations of 25%, 50%, and 75% delivered at a flow rate of 5 µl/min. (**b**) Differential deflection of 50 M anti-TSPAN7 monoclonal antibody coated cantilever (green) upon injection of diluted plasma solution concentrations of 25%, 50%, and 75% delivered at a flow rate of 5 µl/min. (**c**) Differential deflection upon injection of human serum (dark yellow) against 50 µM anti-albumin antibody and plant protein (black) reference. (**d**) Differential deflection upon injection of human serum against anti-albumin antibody 1(dark red), anti-albumin antibody 2 (dark blue) and anti-albumin antibody 3 (red) and 50 µM anti-plant antibody coated cantilever (black) as reference. In (**a**-**b**), the grey shaded area represents the different dilutions of serum and plasma with PBS and (**c**-**d**), The grey shaded area represents the injection of PBS (lasting for 1–3 min), to establish a baseline. The results confirm selective and high-sensitivity detection of serum proteins in a simple and yet robust detection system from a complex biological background.

**Fig. 6:**
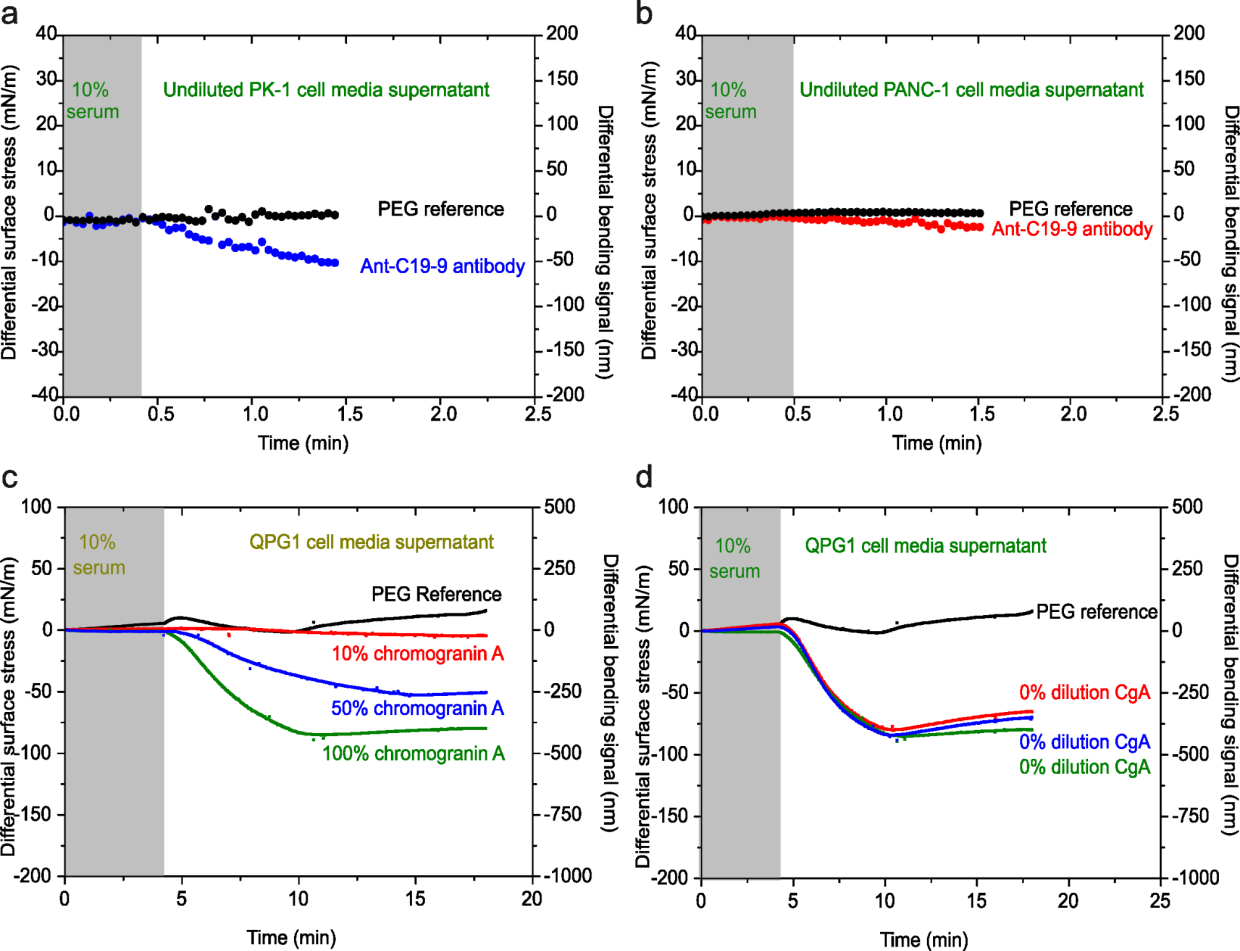
Investigating the biological noise and specificity of biomarker detection. (**a**) Differential deflection of 50 M anti-CA19-9 antibody coated cantilever (blue) upon injection of undiluted PK-1 cell supernatant at 5 µl/min. (**b**) Differential deflection of 50 M anti-CA19-9 antibody coated cantilever (red) upon injection of undiluted PANC-1 cell supernatant at 5 µl/min. (**c**) Differential deflection upon injection at 5 µl/min of serially diluted QPG-1 cell supernatant in PBS at 10% (red), 50% (blue) and 100% (green) against 50 µM anti-CgA antibody and PEG (black) as reference. (**d**) Differential deflection upon injection at 5 µl/min of undiluted QPG-1 cell supernatant (blue), (red) and (green) against 50 µM anti-CgA antibody coated cantilever and PEG (black) as reference. In (**a**-**b**), The grey shaded area represents the injection of PBS (lasting for 0.5–1 min), to establish a baseline. and (**c**-**d**), The grey shaded area represents the injection of 10% serum (lasting for 3–5 min), to establish a baseline. The results confirm selective and high-sensitivity detection of protein biomarkers from serum fluid which represent a complex biological background.

**Fig. 7:**
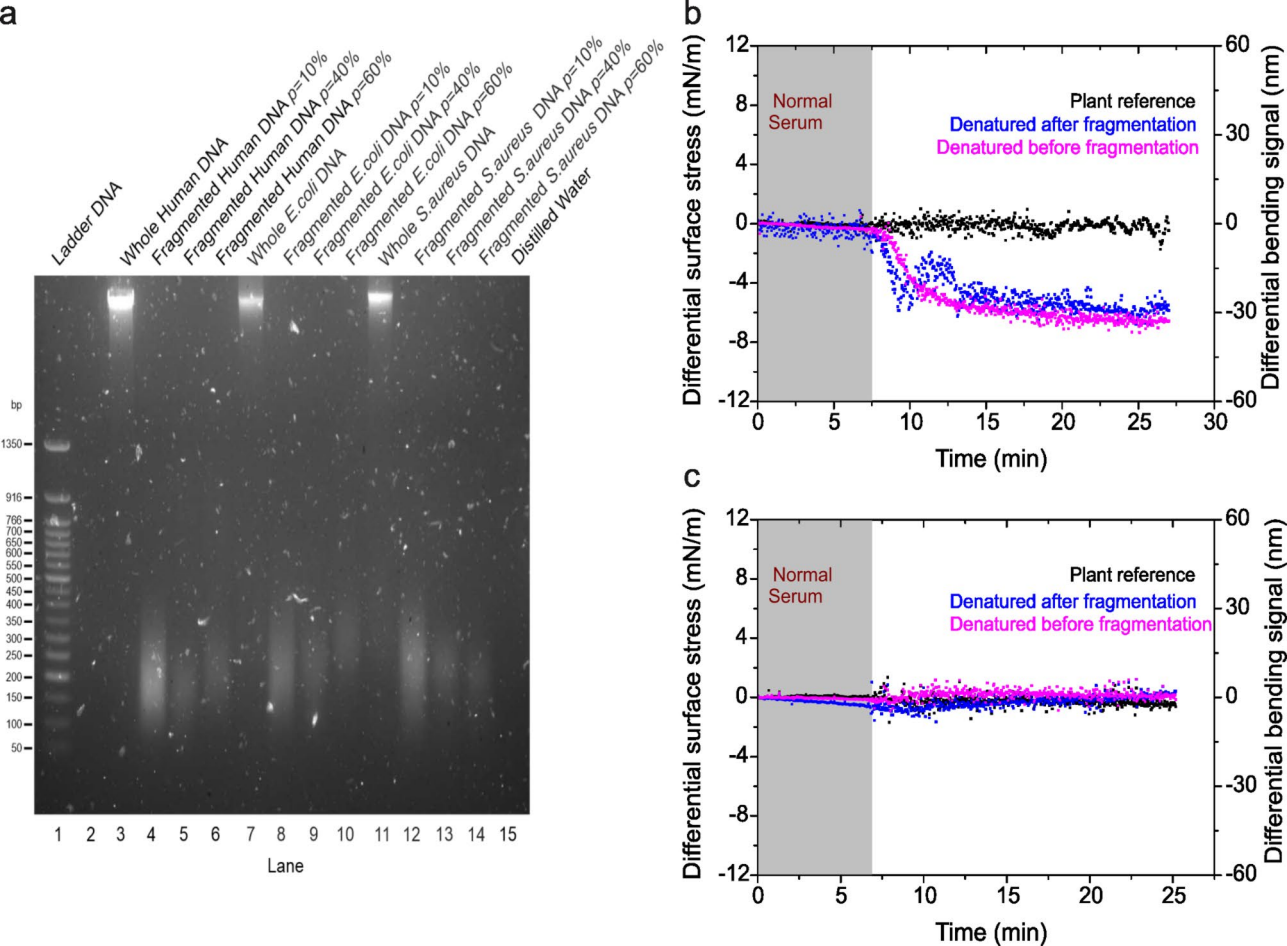
Multiplexed detection of cell-free DNA (cfDNA). (**a**) The cfDNA extracts from human pancreatic cells and bacterial cells. Lane 1, marker DNA ladder; lane 2, empty; lane 3, whole human DNA without fragmentation; lane 4, fragmented human genomic DNA at 10% incident power; lane 5, fragmented human genomic DNA at 40% incident power; lane 6, fragmented human genomic DNA at 60% incident power; lane 7, whole *E*.*coli* (Gram-negative bacteria) genomic DNA without fragmentation; lane 8, fragmented Gram-negative bacteria genomic DNA at 10% incident power; lane 9, fragmented Gram-negative bacteria genomic DNA at 40% incident power; lane 10, fragmented Gram-negative bacteria genomic DNA at 60% incident power; lane 11, whole *S*.*aureus* (Gram-positive bacteria) genomic DNA without fragmentation; lane 12, fragmented Gram-positive bacteria genomic DNA at 10% incident power; lane 13, fragmented Gram-positive bacteria genomic DNA at 40% incident power; lane 14, fragmented Gram-positive bacteria genomic DNA at 60% incident power and lane 15, distilled DNA free water for control measurement. (**b**) The differential surface stress-versus-time data recorded for proxy bacterial sequence surface construct after delivery of 10 ng/µl whole Gram-negative genomic-derived cfDNA denatured before fragmentation (magenta curve) and 10 ng/µl whole Gram-negative genomic-derived cfDNA denatured after fragmentation (blue curve). (**c**) The differential surface stress-versus-time data recorded for bacteria sequence surface construct after delivery of 10 ng/µl whole human genomic-derived cfDNA denatured before fragmentation (magenta curve) and 10 ng/µl whole human genomic-derived cfDNA denatured after fragmentation (blue curve). In (**b**,** c**), The differential reference signal using plant based DNA probe is shown in black, the differential measurements were acquired in a complex liquid media; 10% human blood serum plasma diluted in 1 M NaCl sodium citrate buffer (SCC), pH = 7.4 and the grey shaded area represents the injection of serum solution (lasting for 6 min), to establish a baseline. The results confirm that the selectivity and sensitivity was not adversely affected by plasma serum and, thus nanomechanical systems provide a basis for biological probes without the logistical demands for multistep sample extraction, amplification, or labelling.

To optimise biorecognition, we implemented the following strategies: (1) simultaneously attaching probe sequences to both the top and bottom surfaces of the sensing element to generate a measurable net differential stress, (2) incorporating a nonspecific plant-based probe construct to control for specific molecular recognition and produce distinct signals in the presence of biomarker signatures, and (3) designing a probe construct where signal responses were statistically validated using One-way ANOVA followed by Bonferroni’s post-hoc test (Supplementary Fig. 1). For microbial biomarkers (Figs. 2, 3, 4 and 5), functionalisation of the probe molecules was achieved using self-assembled monolayers (SAMs) consisting of (i) oligonucleotide sequences specific to Gram-negative bacterial species, (ii) oligonucleotide sequences specific to Gram-positive bacterial species, and (iii) oligonucleotide sequences that simultaneously target both Gram-negative and Gram-positive species in real-time detection. For protein biomarkers (Figs. 6 and 7), the nanomechanical cantilever arrays were functionalised on both sides with SAMs of (i) anti-TSPAN7 monoclonal antibody, (ii) anti-CA19-9 monoclonal antibody, and (iii) anti-human albumin monoclonal antibody. We employed multiple chips, each consisting of eight rectangular silicon cantilever arrays, with one side coated with a thin gold film (Figs. 2, 3, 4, 5, 6, and 7). Each chip contained six cantilever biosensors for ligand detection and two reference sensors. The simultaneous functionalisation of the cantilever arrays was achieved by immersing both sides of each sensing element in micro-capillary glass tubes containing 8 µL of SAM solution with capture molecules (DNA probes or antibodies), along with reference SAMs of polyethylene glycol (PEG) and plant-derived agents.

In our previous studies^27^, we demonstrated that a significant contribution from the silicon (Si) underside surface, comparable to that from the gold (Au) top surface, is observed only when SAM concentrations reach or exceed 2000 µM, assuming the silicon surface is not passivated. By using SAMs of capture molecules and reference coatings at a concentration of 50 µM, which is significantly lower than 2000 µM, we were able to optimise the probe constructs without the need for silicon underside surface passivation. We applied this approach as a model for testing the effectiveness of biorecognition events and for developing a realistic in vitro assay to detect inflammation-promoting features, with potential applications across various disease contexts. The corresponding sensitivity and specificity were assessed by validation against clinical qPCR, ensuring its immediate relevance for clinical use.

## Results

### Designing nanomechanical detection for bacterial proinflammatory biomarkers

To create arrays of nanomechanical sensing elements (Fig. 1), analogous to the pattern recognition receptors found on immune cells, we decorated the sensing elements with probe sequences designed to identify highly conserved genomic regions of bacterial species. When a probe interacts with a ligand or antigen, they form a bound probe-ligand or probe-antigen complex. This complex in turn, influences the underlying surface mechanics, causing the sensing element to bend. The bending is induced by the stress in the probe construct from the bound complex and correlates with the amount of bound ligand/antigen, thus providing a signal response for quantitative monitoring of disease biomarkers.

To evaluate the detection of microbial cfDNA, we first simulated model bacteria using a universal probe construct designed for the conserved bacterial 16 S rRNA gene. This approach allows the detection of the total bacterial load in diverse sample types. Notably, the complementary cfDNA derived from the 16 S rRNA gene ranged between 21 and 29 base pairs (bp). The measurement was performed in a liquid chamber with a volume of 80 µL, utilizing a pumping system (GENIE, Kent Scientific) to regulate the exchange of various solutions and flow rates between 5 µL/min and 30 µL/min. The liquid flow facilitated the exchange of solutions and ensured a steady mass transport of ligands, leading to the saturation of the sensor response and achieving a steady-state equilibrium for each concentration.

Before injecting a specific ligand or antigen solution (Figs. 2, 3, 4, 5, 6, and 7), serum or PBS (0.1 M, pH 7.4) solutions were introduced for durations ranging from 1 to 7 min to establish background signals. To produce measurable and consistent signals, we integrated internal references for a more precise differential readout, aiming to reduce the impact of thermal drift and nonspecific binding effects. This is because it is widely accepted that the absolute bending signal can be influenced by both specific and nonspecific factors^25^. Nonspecific influences include variations in temperature, refractive index, interactions between background and specific solutions, and the silicon oxide underside of the nanomechanical cantilever biosensors.

For the detection of microbial signatures, we performed differential measurements by subtracting the bending signals of reference SAMs of probe constructs based on a plant-derived agent from the microbial-specific probe signals. Similarly, for protein biomarkers, we subtracted the bending signals of reference SAMs of PEG or plant proteins from the monoclonal antibody-coated sensor signals (see the Methods Section and Supplementary Information). The exchange of various solutions, including microbial DNA solutions (ranging from 1 fM to 4 µM), normal human blood serum/plasma (10–100%), cell media supernatant (10–100%), and Glycine-HCl Buffer (0.1 M, pH 3.0) regeneration solutions, was achieved using an automated valve and a pumping system.

To investigate the recognition capabilities of the microbial cfDNA candidate regardless of the subtype, we first injected into a measuring chamber a solution containing 0.1 µM universal bacterial cfDNA fragments against a universal probe construct. The results, summarised in Fig. 2a (green curve), demonstrate strong binding specificity, as indicated by a bending response of 12 nm. In contrast, the biosensor coated with probe constructs based on a plant-derived agent showed a negligible signal response (Fig. 2a, black curve). The downward bending response in Fig. 2a indicates the presence of compressive stress, attributed to repulsive electrostatic and steric interactions from the sugar-phosphate backbone of DNA bases and their associated counter ions. To understand the uniformity of the recognition process by the universal probe construct, the sample solution was reconstituted using cfDNA fragments of Gram-positive bacteria at a fixed concentration of 0.1 µM, matching the experimental conditions for the universal bacteria. The recognition event produced an equilibrium differential signal of 10 nm (Fig. 2b, magenta curve), which is comparable to the 12 nm bending signal observed for the universal bacteria (Fig. 2a, green curve). However, the reference probe constructs based on plant-derived agents exhibited negligible bending, as shown in Fig. 2b (black curve).

Next, we aimed to determine if the recognition of Gram-negative bacteria would produce a consistent bending response due to stress in the universal probe construct. For these experiments, we reconstituted the sample solutions with cfDNA fragments of Gram-negative bacteria, maintaining a concentration of 0.1 µM to match the conditions used for both universal and Gram-positive bacteria. Figure 2c (orange curve) displays the equilibrium differential bending signals, indicating a response of 11 nm. This response is consistent with the 10 nm observed for Gram-positive bacteria (Fig. 2b, magenta curve) and the 12 nm recorded for universal bacteria (Fig. 2a, green curve). The reference probe construct-coated cantilevers based on plant-derived agents, as expected, show no bending response against cfDNA fragments of Gram-negative bacteria. These results collectively demonstrate a strong correlation between Gram-negative and Gram-positive bacteria, confirming that the alterations in surface mechanics are closely associated with the universality of bacterial detection, irrespective of the subtype.

To provide a broader context for our findings, we employed a model highlighting the substantial impact of gut microbiota on the host during both homeostasis and disease^31^. In this model, the diversity of bacterial engraftment represents an imbalance in the total microbiome community. The significance of this effect becomes apparent when alterations in the composition of the microbiome ecosystem occur, resulting from the gain or loss of community members or changes in the relative abundance of microbes. As a result, we optimised the conditions for relative abundance and expression to establish an ecosystem with varying proportions of Gram-negative and Gram-positive bacteria. The primary objective of this bacterial engraftment study is to establish a proof of concept for analysing signal responses in relation to the relative abundance of Gram-negative and Gram-positive bacteria within the gut microbiota.

Accordingly, we performed a series of binding analyses using a universal probe construct. We varied the cfDNA proportions to 10%, 50%, and 90% for both Gram-negative and Gram-positive bacteria, while keeping the total cfDNA concentration constant at 0.1 µM. This approach ensured that the overall bacterial load remained consistent with the experimental conditions described in Fig. 2a-c. The differential measurements, as shown in Fig. 2d, revealed the surface stresses induced by specific interactions between cfDNA and the universal probe construct. Upon the injection of 0.1 µM cfDNA fragments from a mixture of Gram-negative and Gram-positive organisms, the differential surface stress signals for 10% Gram-negative (black curve), 50% Gram-negative (blue curve), and 90% Gram-negative (red curve) were 10 nm, 11 nm, and 12 nm, respectively. In contrast, the probe constructs based on plant-derived agents exhibited negligible signal responses, with values for 10% Gram-negative (black curve), 50% Gram-negative (blue curve), and 90% Gram-negative (red curve) indistinguishable from zero. Interestingly, as shown in Fig. 2d, the mechanical response does not scale proportionally with increasing cfDNA concentrations for either Gram-negative or Gram-positive bacteria. Similarly, when Gram-negative and Gram-positive bacteria are balanced at a 50% ratio, the signal response becomes nearly indistinguishable. Notably, the signal values remained consistent within the margin of experimental error. Therefore, under these experimental conditions, the signal response is comparable, regardless of whether Gram-negative or Gram-positive bacteria predominate. This universality of induced stress implies that the stress transduction is independent of the collective phenomenon of individual bacterial subtypes, provided the binding sites are occupied under steady-state equilibrium conditions. As a result, our findings offer a novel approach for the rapid detection of bacteria within the diverse and complex microbial ecosystem of the gut microbiota. This approach could be valuable for initiating prophylaxis as a precautionary measure to prevent infection without the need for organism-specific treatments. However, it is important to note that the universal probe construct is purely phenomenological. It can only indicate the presence or absence of bacterial insults but cannot accurately identify the bacterial subtype or determine the most effective course of action to restore homeostasis in the microbiome ecosystem.

## Nanomechanical profiling of different bacterial subtypes

With the stress signal remaining consistent regardless of whether the dominant subtype is Gram-negative or Gram-positive bacteria (Fig. 2d), our next objective was to assess whether nanomechanical detection could distinguish between different bacterial subtypes. The classification of multiple bacterial cfDNA fragments in patient samples is crucial for enabling accurate differential diagnosis across various clinical contexts. This is especially significant in patients following aggressive chemotherapy regimens, where a notable increase in microbial counts could indicate damage to the intestinal barrier. Precise detection of microbial counts and identification of specific cases are essential not only for recognising patients at risk of neutropenic fever and sepsis but also for providing critical insights to guide organism-specific treatments.

To ensure the specificity of bacterial detection, we investigated the probe constructs targeting Gram-negative and Gram-positive bacterial species for measurement, along with a plant species probe construct to serve as a reference. Initially, the Gram-negative species probe construct was exposed to 4 µM cfDNA fragments from Gram-negative organisms to evaluate its ability to detect these organisms. The examination of the induced stress by the Gram-negative probe construct-biofunctionalised sensing element showed an equilibrium differential bending response of 33 nm (Fig. 3a, orange curve). Conversely, the probe constructs utilizing plant-derived agents exhibited a negligible mechanical response (Fig. 3a, black curve). The significant mechanical response observed when the Gram-negative probe construct is exposed to cfDNA fragments from Gram-negative organisms indicates a potential specific nanomechanical recognition of this bacterial subtype.

To verify the specificity of nanomechanical recognition for cfDNA fragments from Gram-negative organisms, we conducted competitive assays using a mixture of cfDNA fragments from both Gram-negative and Gram-positive organisms, with a total concentration fixed at 0.8 µM. Figure 3b illustrates the results, showing a stable baseline when 10% serum diluted in PBS is injected against the probe construct in the absence of the target sample. Upon exposure to the sample, the Gram-negative species probe construct promptly responded by bending downwards, reaching a stable equilibrium differential compressive bending signal of 25 nm (Fig. 3b, orange curve). In contrast, for the Gram-positive organisms, no discernible effect on stress transduction was observed (Fig. 3b, magenta curve). The bending response was indistinguishable from that of the plant reference (Fig. 3b, black curve). These observations suggest that the mechanical response is significantly influenced by the specificity of the transduction mechanism of the probe–ligand interactions. Specifically, for the Gram-negative species probe construct, the mechanical response is 25 nm when both cfDNA fragments from Gram-negative and Gram-positive organisms are fixed at 0.8 µM (Fig. 3b, orange curve). This response is twice as large as that of the universal probe construct, which exhibited a mechanical response of 12 nm in a competitive environment with a total concentration fixed at 0.1 µM cfDNA fragments from both Gram-negative and Gram-positive organisms (Fig. 2d, red curve).

Intrigued by the specificity of the biorecognition of the probe construct linked to Gram-negative species (Fig. 3a, b), we hypothesised that the probe construct linked to Gram-positive species could effectively and specifically identify Gram-positive organisms. To test this hypothesis, we initially injected 1 µM cfDNA fragments specific to Gram-positive organisms. Notably, the results in Fig. 3c (magenta curve) show that the induced stress response for the Gram-positive probe construct was 40 nm, which is greater than the response of the Gram-negative probe construct to cfDNA fragments from Gram-negative organisms at a fixed concentration of 4 µM, where the equilibrium differential bending response was 33 nm (Fig. 3a, orange curve). The reference plant probe construct-coated cantilevers exhibited an expected negligible bending signal response (Fig. 3c, black curve).

To further verify the specificity of nanomechanical recognition for cfDNA fragments from Gram-positive organisms, we conducted assays in a competitive environment, where the samples contained both Gram-negative and Gram-positive species, with a total concentration fixed at 4 µM. In the serum solution without the sample, a stable baseline was observed. However, upon sample injection, the biosensor elements coated with the Gram-positive probe construct exhibited a rapid downward bending, stabilising at an equilibrium compressive bending signal of 40 nm (Fig. 3d, magenta curve). In contrast, the probe constructs specific to Gram-positive species showed a negligible signal response to Gram-negative organisms (Fig. 3d, orange curve), consistent to the response observed for the nonspecific plant-based probe construct used as a reference (Fig. 3d, black curve), when exposed to 4 µM cfDNA fragments from both Gram-negative and Gram-positive organisms, demonstrating specific molecular recognition. In addition, we find that the induced stress response for the Gram-positive probe construct differs from that of the universal probe construct (Fig. 2b, magenta curve).

The consistency in mechanical response observed between standalone and competitive assays is expected, as the net binding energy of ligand-receptor interactions—including hydrogen bonding, electrostatic forces, hydrophobic effects, and van der Waals interactions—remains unchanged, regardless of whether the cfDNA fragment originates from Gram-negative organisms alone or in combination with Gram-positive organisms^26–28^. This holds true if we assume that, in a competitive environment, these fragments do not dimerise in solution or on the surface-immobilized probe constructs. Therefore, the surface mechanics are significantly influenced by the specificity of the probe–ligand interaction transduction mechanism. This high reproducibility and specificity confirm the surface construct’s functionality in accurately identifying signatures associated with different bacterial subtypes in a single sample. Furthermore, the presence of distinct bending responses in competitive assays with both Gram-negative and Gram-positive species illustrates how multiplexed detection of signature organisms with high selectivity and sensitivity can be achieved to unambiguously distinguish the quantity and identity of the organisms. These findings strongly suggest that nanomechanics is closely related to the efficacy of the receptor or capture molecules immobilized on the surface, conclusively confirming the excellent specificity for detecting cfDNA fragments from different bacterial subtypes. Accordingly, this approach demonstrates that simultaneous quantitative monitoring and identification of diverse bacterial subtypes within the microbiome environment can be achieved in a single step, facilitated by using a species-specific surface probe construct.

We then evaluated the effect of cfDNA fragment concentration on direct mechanical assays by monitoring the stress response following injection of cfDNA fragments from Gram-negative organisms at concentrations of 4 µM (Fig. 3a, orange curve) and 0.8 µM (Fig. 3b, orange curve) against Gram-negative probe constructs. The results demonstrated an increase in stress response proportional to the rising cfDNA concentration. To determine if this trend persists across microbial types, we analysed the mechanical signals produced by cfDNA fragments from Gram-positive organisms at concentrations of 1 µM (Fig. 3c, magenta curve) and 4 µM (Fig. 3d, magenta curve) against Gram-positive probe constructs. The differential signal response remained at 40 nm for both concentrations, suggesting that increasing cfDNA concentration beyond 4 µM does not further enhance the signal response, indicating surface saturation. These findings suggest that a defined set of general guidelines could enable the development of accurate, real-time tests to assess the microbiome’s impact on adaptive immune-mediated proinflammatory gastrointestinal and extraintestinal diseases. During the initial stages of epithelial barrier damage, proinflammatory marker levels in the circulatory system are likely to fluctuate significantly, resulting in pre-saturation signal responses. At advanced stages of mucosal epithelium damage, however, proinflammatory markers are expected to reach high concentrations, producing invariant signal responses due to saturation. Distinct signal response profiles, such as those shown in Fig. 3a-d, may therefore be tailored to model each phase of disease for effective staging and progression monitoring.

## Time-resolved dynamics of ligand quantitation: selectivity and sensitivity

The critical aspects for developing a viable diagnostic technology are sensitivity and specificity. Sensitivity, in this context, refers to the lowest detectable limit (LOD) of a disease marker that can generate a measurable signal. To evaluate sensitivity and specificity, we monitored changes in signal response over a 30-minute period at different dilutions of microbe-associated samples, using both Gram-negative and Gram-positive probe constructs. Figure 4a illustrates representative time- and concentration-dependent data at 30 µl/min, showing a well-defined signal increase with alternating increases in concentrations. Here we observed that lower microbial DNA concentrations (1 fM to 1 nM) required approximately 50 to 60 min to reach thermodynamic pseudo-equilibrium. In contrast, higher microbial DNA concentrations (100 nM to 4 µM) achieved equilibrium conditions in less than 10 min. The negligible response observed in the in-situ reference based on plant-coated nanomechanical cantilever sensors, indicates resistance to biomolecule adsorption on the surface, thereby ensuring specific binding.

Further, we performed nanomechanical quantitative assays under steady-state equilibrium conditions, where the relative microbial DNA concentrations and the extent of microbial DNA binding were quantitatively monitored by measuring the differential stress changes as a function of increasing concentration using serially diluted microbial DNA concentrations while keeping the flow rate constant at 30 µl/min. After each injection, we regenerated the sensor surface using glycine-HCl buffer solution to ensure that binding sites were available for subsequent injections. This process continued until a plateau was reached (Fig. 4b-d). In our previous studies^25^, we determined that the number of capture probe molecules was ~ 1 × 10^11^per sensor, with each molecule covering an area of ~ 44 Å^2^. We also demonstrated that achieving around 10% surface coverage was necessary to establish connectivity between chemically transformed regions, resulting in an observable signal response. The data in Fig. 4b-d, along with calculations showing that microbial DNA at a 4 µM concentration contains an estimated ~ 2.4 × 10^18^ target molecules, provide strong evidence that all accessible binding sites on the sensor surface are fully occupied. This suggests an efficient exchange between solution target molecules and binding sites on the sensor surface. Additionally, the pumping system effectively regulates this exchange process, ensuring the removal of any excess target molecules.

To demonstrate the ultimate sensitivity and to support our hypothesis that detection sensitivity and dynamic range are significantly influenced by the detailed design of the surface construct, we measured the binding interactions of cfDNA fragments from Gram-positive organisms with Gram-positive probe constructs. The data in Fig. 4b reveal a detection sensitivity of 9 fg/µL (~ 250 fM) of *S*. *aureus* (Gram positive) DNA, equivalent to detecting approximately 30 bacterial cells. The sensitivity reported for antibiotic resistance genes in Gram negative bacteria, particularly *Pseudomonas aeruginosa*, is 10 fg/µL total RNA. For resistance genes in Gram-positive bacteria, such as *Enterococcus faecium*’s vanD, the sensitivity reported is 1 fg/µL total RNA^32,33^. The agreement between these findings and our measurements strongly indicates the critical role of nanomechanical sensors in achieving ultrasensitive detection of infectious agents. Moreover, the observed dynamic range exceeds that of qPCR assays^34,35^, which have reported detection limits of 10 pg/µL, making our method approximately 10,000 times more sensitive. The improved sensitivity and dynamic range of our approach were further confirmed by using two additional agents designed to recognise a broad spectrum of bacteria (Fig. 4c, d). These results collectively suggest that the intricate surface probe construct not only enhances the limit of detection and dynamic range but also governs the reproducibility and specificity of ligand detection. This enhancement is crucial for the effective diagnosis of sepsis, where rapid and ultrasensitive detection of microbial signatures is critical.

Next, we explored quantitative selectivity, assessing how well the probe-ligand pairings match in terms of complementarity. This measurement provides insight into the surface free energy. In this case, we first used Eq. (1), whose detailed derivation has been reported previously^26^,

1 $$\:\varDelta\:{\sigma\:}_{eq}=\varDelta\:{\sigma\:}_{max}\left(\frac{{\left[ligand\right]}^{n}}{{K}_{d}^{n}+{\left[ligand\right]}^{n}}\right)$$

where σ_*eq*_ corresponds to equilibrium surface stress, σ_*max*_ is maximum stress at full coverage, *n* is the stoichiometric coefficient and *K*_*d*_ is the surface equilibrium dissociation constant. By fitting the experimental data using Eq. (1), we extracted a *K*_*d*_ value of 55.3 × 10^−9^ M for Gram-negative species and 48.3 × 10^−9^ M for universal model bacterial species. The corresponding σ_max_ generated are 27.6 ± 3 mNm^−1^ and 34.6 ± 3 mNm^−1^ with the values of *n* = 0.6 in both cases. For Gram-positive species, the *K*_*d*_ value is 33.4 × 10^−9^ M and the σ_max_ is 42.6 ± 3 mNm^−1^, while *n* is = 0.5. To compare the Gibbs free energy (ΔG) of surface reactions for different probe constructs, we initially assumed that at equilibrium, and at a constant temperature and pressure, the changes in $\triangle\,G_{ex}$ are zero. We then calculated the ΔG of reactions by using the extracted *K*_*d*_ values. Specifically,

2 $$\:\varDelta\:G=RTln\left({K}_{d}\right)$$

where *R* is the ideal gas constant and *T* is the absolute temperature. Using Eq. (2), the Δ*G* was calculated as −42 kJ mol^−1^ for universal probe construct and − 42 kJ mol^−1^ for Gram-negative species probe construct. Similarly, the calculated Δ*G* for Gram-positive species probe construct is −42.6 kJ mol^−1^. We also evaluated alternative techniques, including surface plasmon resonance (SPR) and the cantilever method, to validate our findings for the selected markers. Consistent with previous studies, Gibbs free energy values of Δ*G* = −41.4 kJ mol^−1^were observed using the cantilever method^36^, and Δ*G* = −43.4 kJ mol^−1^with the SPR technique^37^. This strong agreement underscores the specificity and sensitivity of nanomechanical recognition for cfDNA fragments, achieved through a straightforward yet robust detection system within a complex biological matrix such as serum. For precise error analysis in the surface stress data, we calculated the standard deviation of measurements from three separate sensor chips (see Supplementary Section ‘Conversion of the bending signals into differential surface stress’). This approach enabled an accurate assessment of binding kinetics and equilibrium characteristics in the molecular interactions, enhancing the reliability and robustness of our results.

## Influence of blood serum on the specificity of nanomechanical biomarker detection

The bending signal is susceptible to noise from both the instrument and biological samples, with factors like interference between the incident and reflected laser light contributing to instrument noise. Maintaining a high signal-to-noise ratio involves minimising room light, using a high voltage supply for enhanced laser intensity, and addressing noise from biological fluids, primarily from non-specific interactions. Interference in biomolecular recognition within blood can be caused by various factors. For example, serum contains a wide range of proteins and biomolecules that may non-specifically bind to sensor surfaces, leading to false-positive signals and decreased specificity. Additionally, serum components can obscure target molecules, making accurate detection challenging. Competition for binding sites between serum proteins and target molecules can further reduce binding specificity. Moreover, serum components may contribute to background noise in sensor signals, complicating the differentiation between specific and nonspecific signals. Therefore, evaluating the impact of serum/plasma on the specificity of biomarker detection is crucial for ensuring accurate diagnostics and clinical decision-making.

To define a signal response range in human blood samples where biological noise does not impair biomarker detection specificity, we prepared 25%, 50%, and 75% dilutions of whole human serum and plasma from healthy donors using PBS. Anti-TSPAN7 was selected as the sensing molecule for its ability to detect TSPAN7, a transmembrane protein with various cellular functions that can be released into serum or plasma either as a soluble protein or within extracellular vesicles or exosomes^38^. This setup serves as a robust model to differentiate specific biomarker interactions from biological noise.

To evaluate this, we injected varying concentrations of diluted serum across nanomechanical sensors coated with anti-TSPAN7 monoclonal antibodies at a flow rate of 30 µL/min for 50 min. In Fig. 5a (blue curve), we illustrate the peak-to-peak noise variations in serum, with the grey-shaded region representing noise level fluctuations across conditions. For comparative analysis, similar measurements were performed with plasma at the same dilutions of 25%, 50%, and 75%, as shown in Fig. 5b (green curve). Our results reveal that plasma exhibits higher peak-to-peak noise variations than serum, likely due to laser scattering caused by cellular debris. Liquid exchange and mixing challenges occasionally led to overlap between regions, complicating the resolution of specific signals. Despite increased noise in plasma, the differential signal from anti-TSPAN7-coated arrays was preserved, showing negligible bending response, which confirms the absence of detectable TSPAN7 in serum samples. These findings underscore the minimal influence of nonspecific interactions and biological noise on the specificity of nanomechanical detection. Furthermore, high peak-to-peak noise can be effectively reduced through external software filtering without altering the underlying data.

We next investigated whether the negligible bending response in serum, as shown in Fig. 5a, b, could be due to nonspecific interactions. To examine this, we used a nanomechanical sensor coated with anti-albumin to detect serum albumin, with a plant protein-coated sensor serving as a reference. This setup was selected because serum albumin fluctuations are often indicative of liver cirrhosis severity and systemic inflammation^39^. Therefore, developing a rapid and accurate sensor for quantitative albumin monitoring could be highly valuable in healthcare. Figure 5c shows the bending signals from arrays featuring an anti-albumin sensor (light green curve) alongside the plant-based reference (black curve). Following serum injection, the anti-albumin sensor produced a bending response that stabilised at a differential equilibrium of 45 nm after a 10 min injection at 30 µL/min.

To evaluate the reproducibility of nanomechanical signals in serum, we conducted measurements using three anti-albumin-coated sensors, resulting in bending signals of 50 nm (wine curve), 55 nm (blue curve), and 57 nm (red curve) as shown in Fig. 5d. The consistency of within-array measurements, alongside greater variability between arrays, aligns with our prior findings^25^. This specificity of the nanomechanical response is underscored by comparing the absence of albumin recognition in serum when using the anti-TSPAN7 sensor to the response observed in the in-situ reference (Fig. 5a). Additionally, to compare the magnitude of stress variations in serum, we analysed the mechanical responses shown in Fig. 5c, d. These responses reveal notable variations in bending values that fall within the experimental error margin, confirming that the serum-albumin interactions responsible for cantilever bending are distinct from the nonspecific interactions observed in Fig. 5a. Consequently, this confirms that the negligible bending response observed in Fig. 5a, b is indeed attributable to nonspecific interactions. This suggests that enhancing the efficacy of nanomechanical signals is possible by optimising the intrinsic complementarities between ligands/antigens and surface probe/capture molecules.

To further validate our approach and confirm our hypothesis that specificity is significantly influenced by the detailed design of the surface construct, we conducted additional experiments using an anti-CA19-9 IgG sensor, with PEG serving as an in-situ reference. We used CA19-9 because it is an FDA-approved marker commonly employed in PDAC diagnosis. We utilized PK-1 supernatant from cell culture media, as CA19-9 is highly expressed in PK-1 cells but not in PANC-1 cells^40^. The injection of undiluted PK-1 cell media supernatant exhibited a bending response, stabilising at a differential equilibrium response of 80 nm (Fig. 6a, blue curve). In contrast, we observed zero differential stress for the reference PEG-coated cantilevers (Fig. 6a, black curve). As an additional control, we utilized cell media supernatant from PANC-1 cells, which do not express CA19-9. The injection of undiluted PANC-1 cell media supernatant showed a minimal mechanical response (Fig. 6b, red curve). This response, indistinguishable from that of the PEG-coated reference (Fig. 6b, black curve), further confirms that the detected signal specifically results from interactions between CA19-9 and anti-CA19-9 IgG.

To verify the specificity and performance of mechanotransduction during nanomechanical recognition events, we utilized an anti-CgA sensor, with PEG serving as an in-situ reference. The anti-CgA was selected for its anticipated high specificity and sensitivity in binding CgA. Figure 6c displays the results after injecting CgA from unfiltered QGP1 cell media supernatants, diluted in PBS to concentrations of 10%, 50%, and 100% CgA. The bending response demonstrated a scaling effect with increasing concentrations, yielding equilibrium differential signals of 50 nm (red curve), 250 nm (blue curve), and 375 nm (green curve), respectively.

We then repeated the experiment using 100% CgA across three separate nanomechanical sensors (Fig. 6d). The measured nanomechanical responses were 350 nm (red curve), 360 nm (blue curve), and 380 nm (green curve), resulting in a standard deviation of 10 nm and a mean of 360 nm, underscoring the high precision of nanomechanical sensing. These findings indicate that even when serum protein concentrations significantly exceed those of disease biomarkers, the specificity of the probe constructs, combined with the inherent sensitivity of the nanomechanical sensors, can enhance biorecognition events beyond nonspecific interactions.

## Effects of human and bacteria cell debris on the specificity of nanomechanical detection

The disintegration of bacterial or human cell structures results in cellular debris, which includes cell walls, membranes, cytoplasm, nuclei, and organelles. This debris can compromise the specificity of nanomechanical detection by introducing noise and interfering with signal responses. To evaluate the impact of cellular debris on the specificity of nanomechanical detection in complex samples, we conducted experiments using lysates from viable bacteria and intact human cells. These experiments aimed to demonstrate the potential for detecting microbial signatures as observed in vivo and to test the hypothesis that optimised probe constructs, in conjunction with the inherent sensitivity of nanomechanical sensors, can produce a robust signal for cfDNA detection from human samples or bacterial cells, despite the presence of background noise.

Accordingly, we tested this hypothesis using samples derived from (1) human pancreatic cell extracts, (2) *Escherichia coli* cell extracts, and (3) *Staphylococcus aureus*cell extracts. The evaluation included the capacity to generate random signatures of cfDNA fragments with lengths closely matching clinically relevant sizes^10^. For these experiments, the size distribution of cfDNA fragments ranged from 50 bp to 300 bp (Fig. 7a; lanes 3–14 and Supplementary Fig. 3a). To ensure that any observed cfDNA fragment originated from the experimental samples and was not an artifact, we employed distilled water without cfDNA fragments as a negative control to establish a baseline (Fig. 7a; lane 15).

We utilized 10 ng/µL of denatured microbial cfDNA both before and after fragmentation. To further investigate how detection performance is influenced by nonspecific binding of serum proteins, the microbial cfDNA fragments were reconstituted in human serum, which had a total background protein concentration of 60 mg mL^−1^, approximately 10^8^-fold larger than that of the cfDNA. As shown in Fig. 7b, the bending signals demonstrated a notable similarity in compressive stress, measuring 30 nm (blue curve) for the cfDNA fragments denatured after fragmentation and 33 nm (magenta curve) for those denatured before fragmentation. These findings are consistent with previous results (Fig. 3), which showed that the signal response exhibited a rapid increase within the initial reaction time of less than 10 min, before stabilising at 30 nm. This is because, at a concentration of 10 ng/µL, the cfDNA corresponds to approximately 9 × 10^13^ target molecules, which exceeds the estimated occupancy of the capture probe molecules at around 1 × 10^11^per sensor^25^. At this concentration, all accessible binding sites on the sensor are likely to be fully occupied, as illustrated in Fig. 4b-d. Additionally, the recognition process remained unaffected by the added biological complexity. As expected, the negligible response observed in the in-situ references using a plant sensor during control experiments further confirms the specificity of nanomechanical recognition, even in the presence of cellular debris.

We then investigated how the nanomechanical response is affected when the source of cfDNA originates from humans while using a probe construct based on microbial genes. In these experiments, cfDNA fragments were extracted from human pancreatic cells, exhibiting a size distribution of 50 bp to 300 bp, consistent with the experimental conditions applied to the microbial cfDNA fragments. Figure 7c presents the bending responses, where the blue curve corresponds to cfDNA fragments denatured after fragmentation, and the magenta curve represents those denatured before fragmentation. The results reveal negligibly indistinguishable mechanical signals observed before and after the recognition step for both measurement and control experiments. These findings strongly establish a significant relationship between the magnitude of signal generation and the specificity of nanomechanical recognition. Consequently, this affirms that the inherent sensitivity of nanomechanical sensors, combined with optimized surface constructs, can generate robust signals for detecting cfDNA from bacterial cells, even amidst the complex biological media introduced by human serum.

We next sought to validate the specificity of our probe constructs for detecting Gram-positive and Gram-negative bacterial species. For this purpose, we employed qPCR across various concentration ranges. Our focus was on evaluating analytical specificity, sensitivity, LOD, and reproducibility using qPCR, a technique widely utilized in clinical settings. This approach allowed us to confirm the functionality of the probe constructs in specific nanomechanical assays while ensuring immediate practical relevance and applicability.

**Fig. 8:**
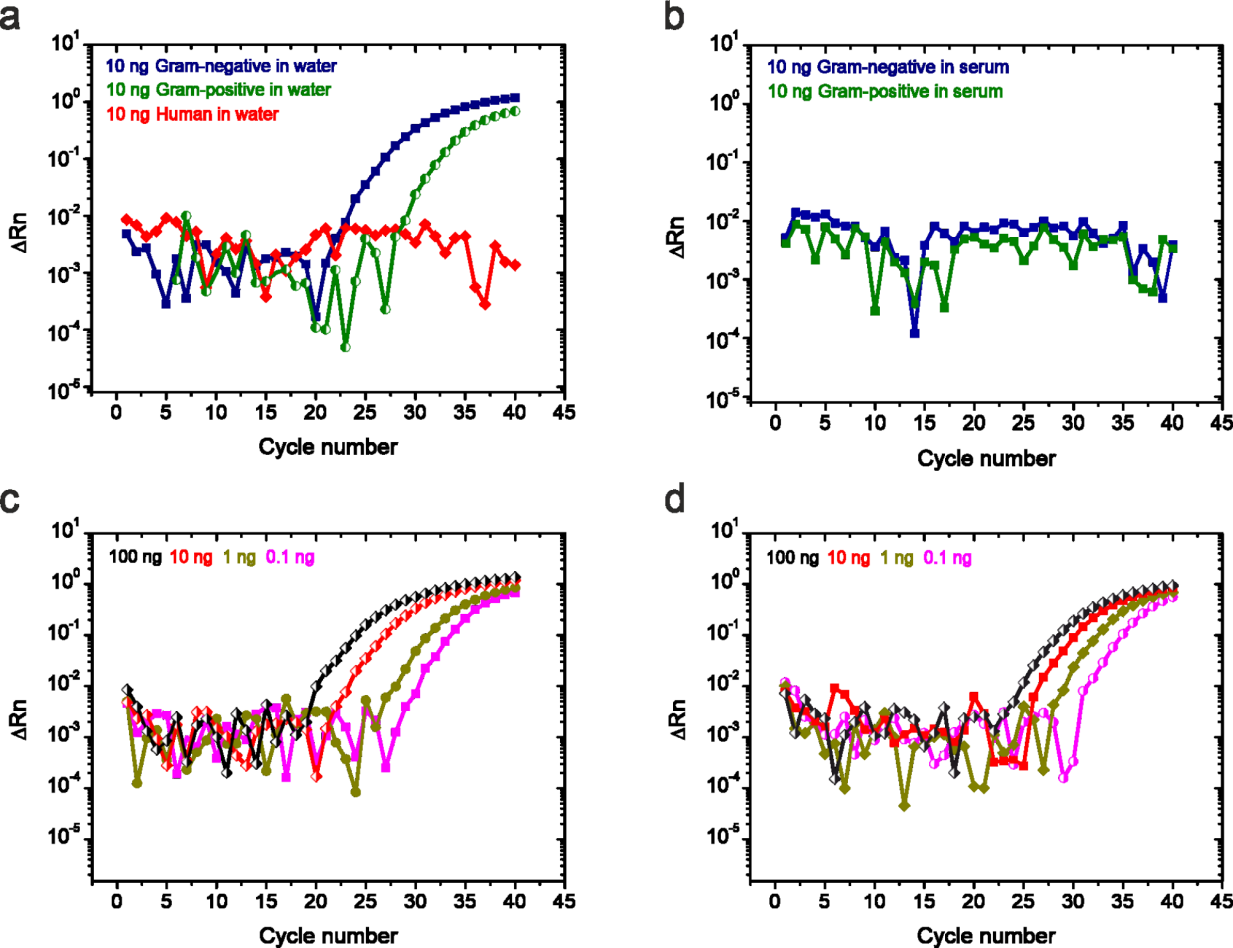
qPCR to inspect the sensitivity and selectivity of the capture agents. (**a**) A qPCR plot showing positive amplification of 10 ng Gram-negative bacteria cfDNA (blue line with solid symbols) and 10 ng Gram-positive bacteria cfDNA (green line with semi-solid symbols) in saline sodium phosphate buffer diluted in DNase-free water showing a growth fluorescent signal phase at around cycle number 25 and 30, respectively. Human cfDNA (red line with solid symbols) showing no amplification as evidence by negligible change in the fluorescent signal in the entire 40 cycles. (**b**) A qPCR plot showing no amplification of 10 ng Gram-negative bacteria cfDNA (blue line with solid symbols) and 10 ng Gram-positive bacteria cfDNA (green line with solid symbols) in human serum showing no amplification as evidence by negligible change in the fluorescent signal in the entire 40 cycles. (**c**) A qPCR plot showing positive amplification of Gram-negative bacteria cfDNA fragments in DNase-free water which gradually decreases as follows; 100 ng (black line with solid symbols), 10 (red line with solid symbols), 1 (dark yellow line with solid symbols) and 0.1 ng (magenta line with solid symbols). (**d**) A qPCR plot showing positive amplification of Gram-positive bacteria cfDNA fragments in DNase-free water which gradually decreases as follows; 100 ng cfDNA (black line with solid symbols), 10 ng cfDNA (red line with solid symbols), 1 ng cfDNA (dark yellow line with solid symbols) and 0.1 ng cfDNA (magenta line with solid symbols). In (**c**,** d)**, The qPCR plot signal shifts to the right on the cycle number as the sample becomes more dilute. We imposed a 40 cycle cut-off threshold because any readings beyond this cycle number was considered unreliable. The data confirm that the surface constructs are highly selective to bacteria, where the instrument detection limit is down to ~ 0.01 ng.

To achieve this, we performed a series of qPCR amplification analyses targeting specific cfDNA fragments from a complex pool of Gram-negative and Gram-positive species derived from pure cultures, which were reconstituted in PBS diluted with DNase-free water. The concentration of cfDNA ([cfDNA]) was consistently maintained at 10 ng/µL to align with the experimental conditions of the nanomechanical assays. Figure 8a presents the results, showing observable changes in amplification cycles: cycle 20 for Gram-negative bacteria (blue curve) and cycle 25 for Gram-positive bacteria (green curve). These changes correspond to the amplification process, indicating a response generated through probe hybridization with its complementary DNA target. In contrast, negative control experiments using cfDNA fragments extracted from human pancreatic cells exhibited no detectable amplification (red curve), clearly demonstrating the ability to distinguish between bacterial and human cfDNA sources.

We further investigated whether the interference from serum impacted the analytical specificity and sensitivity of cfDNA detection in qPCR. The cfDNA fragments from both Gram-positive and Gram-negative bacteria, reconstituted in a total serum protein concentration of 60 mg/mL, showed no observable changes in amplification between cycles 20 and 40 for Gram-negative (blue curve) and Gram-positive (green curve) (Fig. 8b). Comparing the significant signal response observed for microbial cfDNA fragments in a serum concentration of 60 mg/mL during nanomechanical detection (Fig. 7b) with the negligible amplification signal detected by qPCR (Fig. 8b) convincingly demonstrates that the nanomechanical detection system remains unaffected by serum media or additional biological complexities.

To demonstrate the maximum sensitivity achievable with qPCR, we progressively reduced cfDNA concentrations reconstituted in DNase-free water from 100 ng to 0.001 ng. Figure 8c illustrates the results, showing distinct amplification changes for Gram-negative bacteria across cycles 15 to 30 at concentrations of 100 ng (black curve), 10 ng (red curve), 1 ng (green curve), and 0.1 ng (magenta curve). To further assess qPCR sensitivity, we conducted similar measurements for Gram-positive organisms. Figure 8d shows amplification changes across cycles 20 to 30 for concentrations of 100 ng (black curve), 10 ng (red curve), 1 ng (green curve), and 0.1 ng (magenta curve). The limit of detection (LOD) for microbial cfDNA in qPCR was established at 0.01 ng (10 pg), consistent with the detection of bacterial DNA in blood at pretreatment levels, where 250 CFU/mL is reported^34^. The picomolar detection limit for microbial cfDNA using qPCR, as reported here, in contrast to the femtomolar detection limit achieved by the nanomechanical detection system, clearly demonstrates that surface mechanics play a crucial role in enhancing the efficacy of molecular recognition. Further comparative advantages of the nanomechanical detection system over qPCR are summarised in Table 1.

**Table 1:** Comparison of the analysis time and limit of detection of biomarkers between nanomechanical sensors and qPCR.

Description	Nanomechanical sensors	qPCR technique
Time to detection	≤ 5 min	≥ 40 min
Medium	Any including blood serum	DNase-free water
Sample extraction	Not required	Required
Sample labelling	Not required	Required
Limit of dection	Femtogram	Picogram
Sample amplifaction	Not required	Required
Amplicons	No amplicon length	Requires minimum amplification length
Multiplexing ability	High	Low
Portability	Easily portable (handheld)	Localised laboratory setting
Commercial availability	Not yet	In commercial use

## Discussion

Our extensive experimental investigation into cfDNA signatures and secretory proteins across various media complexities has revealed the vital role of complementarity in the transduction between chemical interactions and nanomechanical response. We have demonstrated the ability to detect specific microbial DNA, irrespective of the subtype. These findings underscore how changes in surface mechanics are intricately linked to the universal detection of bacteria, irrespective of subtype. Furthermore, we have validated the specific detection of microbial DNA, showcasing how the nanomechanical response of biorecognition is significantly influenced by the specificity of the transduction mechanism in probe-ligand interactions, despite interference from multiple factors. Additionally, we have illustrated the specific detection of protein markers despite interference from serum/plasma and other background proteins. Moreover, we have shown the specific detection of microbial DNA even after exposure to a serum sample containing human DNA and serum albumin. Consequently, our study affirms that each nanomechanical sensor’s response within a single chip is autonomous, and the specificity of molecular recognition hinges on the complementarity between antigens or ligands in solution and the receptor or capture molecules immobilized on the surface. Therefore, the specificity of molecular recognition, which relies on the complementarity between antigens or ligands in solution, further verifies that microbial cfDNA signatures and secretory proteins can be detected simultaneously on a single biosensor chip.

Our comprehensive experimental study of microbial cfDNA signatures and secretory proteins at clinically relevant concentrations has significant potential to enhance point-of-care screening for inflammatory and cancer-related diseases. Patients with proinflammatory diseases, like liver cirrhosis or sepsis, often face bacterial infections due to compromised innate immunity. These infections exacerbate the already deranged immune response, leading to organ dysfunction. Managing advanced stage proinflammatory diseases is costly and challenging, requiring complex procedures and immunotherapy. To address these challenges and enhance mitigation strategies, accurate and early detection of clinical insults is essential. Our study demonstrates that by leveraging the inherent sensitivity of nanomechanical sensors along with optimised surface constructs, it is possible to detect disease biomarkers at ultra-low quantities, which is crucial for the effective management of sepsis and persistent proinflammatory diseases. Previous methods have utilized a hybrid mechanical element combined with nanoparticle labeling to improve detection limits^22,41^. However, this approach is both expensive and incapable of quantitatively tracking biomarkers across different stages of disease. In contrast, our novel approach overcomes these limitations and provides valuable insights into detecting biomarkers at various stages of disease, which traditional methods would fail to identify.

We hypothesised that utilising nucleotide sequences for optimal diagnostic targets aligned in silico, combined with the inclusion of optimised surface constructs, could offer an inexpensive technological solution. This approach aimed to achieve sub-femtomolar sensitivity and selectivity while addressing the potential limitations of a narrow dynamic range. To confirm this hypothesis, we initially designed three sequence panels targeting bacterial signatures, ensuring high detection sensitivity and accurate bacterial characterisation, a recognised source of inflammation^1–4^. Considering variations in the number of 16 S rDNA sequences in bacterial genomes, novel strategies are required to enhance effective sensitivity for analysing any bacterial source, regardless of relative abundance. A novel surface construct, eliciting the largest nanomechanical response specific to any biorecognition event, as proposed here could be used to detect cfDNA fragments and overcome the challenge of low abundance among plasma proteins. Assessing the statistical significance of signal responses at varying concentrations of the probe construct using one-way ANOVA, followed by Bonferroni’s post-hoc test, may play a valuable role in validating the consistency and sensitivity of the detection system. The mechanical response at 50 µM of probe construct surface biofunctionalization was found to be statistically significant (*p* < 0.001), prompting its implementation in each sensing element to quantitatively monitor microbial signatures while minimising biological noise from the complexity of blood serum samples.

To assess the accuracy of pathogen identification, we conducted a comparative analysis using quantitative qPCR, a commonly employed clinical technique. For validation, bacterial samples were reconstituted in both human serum and DNase-free water. Several key findings emerged from this comparison. First, positive amplification between primers and samples aligned with the nanomechanical response, confirming selective bacterial detection. Second, the nanomechanical measurements demonstrated a remarkable 10,000-fold improvement in detection limit and dynamic range over qPCR assays, largely due to the nanomechanical system’s ability to bypass the multiple processing steps inherent to qPCR and similar amplification techniques. Third, measurements conducted in serum, which contains protein concentrations up to 6 billion times greater than the target markers in blood serum samples, confirmed how surface mechanics play a crucial role in enhancing the efficacy of molecular recognition. While qPCR and related techniques face challenges due to interference from serum proteins, our findings show that the nanomechanical detection system maintains high selectivity, sensitivity, and dynamic range even within complex biological media, unaffected by serum or additional biological complexities.

In summary, we propose that this approach holds significant potential for microbiome sensing applications, as well as for early disease diagnosis and staging. We further suggest that nanomechanical multiplexed detection of multiple biomarkers in real-time could support studies of intestinal permeability in cases where adaptive immune responses are impaired. Additionally, this technology may prove invaluable for evaluating epithelial barrier integrity following intensive therapeutic interventions. The unique advantage of this approach lies in its capacity to deliver direct evidence of bacterial infections associated with chronic diseases, including rheumatoid arthritis, multiple sclerosis, Parkinson’s disease, and Alzheimer’s disease. This capability could enable accurate patient stratification and facilitate rapid clinical decision-making in complex cases involving residual disease. However, to realise its full clinical impact, this approach will require a robust, portable device suitable for point-of-care applications. Therefore, our findings highlight the importance of clinical validation efforts to promote broader adoption of nanomechanical devices across diverse healthcare settings, including emergency and intensive care units, haematology and transplant units, and in resource-limited or remote medical facilities.

In conclusion, we speculate that our approach will play a crucial role not only in microbiome sensing applications but may also play a crucial in early disease diagnosis and disease staging. We further propose the deployment of nanomechanical multiplexed detection for real-time monitoring of multiple markers, could enable interrogation of intestinal permeability in case of breakdown of the adaptive immune response. This could also serve as a versatile medical device to track the structural integrity of epithelial barriers following aggressive therapies. Due to the unique features of this technology, we anticipate its future application in providing definitive evidence of bacterial infection in chronic medical conditions such as rheumatoid arthritis, multiple sclerosis, Parkinson’s, and Alzheimer’s diseases, facilitating precise patient stratification, and enabling rapid decision-making in complex residual disease therapy. Finally, for our approach to be effective in clinical practice, it would require a device that is robust, portable, and suitable for use at the point of care. Therefore, our findings should encourage future efforts toward clinical validation. The implementation of nanomechanical devices will be essential in various settings, including emergency care, intensive care units, haematology/transplant units, and even in resource-limited or remote healthcare facilities.

## Online methods

### DNA sample preparation

Two bacterial cell types were used for cell-free DNA (cfDNA) extracts; Escherichia coli (NCTC 9001, Public Health England) and Staphylococcus aureus (NCTC 8530, Public Health England). Both cell lines were tested for mycoplasma contaminations. Briefly, cells were cultured in Nutrient broth (Sigma Aldrich) at 37 ^◦^C with shaking for 24–48 h. To prepare the cells for cfDNA extraction, the cells were harvested by centrifugation at 5000 g for 5 min. DNA extraction was undertaken using the Qiagen QIAamp DNA Mini Kit (Hilden, Germany) according to the manufacturer’s protocol.

We used human cell-free DNA (cfDNA) from the PANC-1 cell line, acquired from the American Type Culture Collection (ATCC) cell bank. Additionally, cell supernatant was collected from PANC-1, PK-1 and QPG1 cell line in vitro cultures. They were grown in RPMI 1640 media, supplemented with 10% fetal bovine serum, 2 mM per litre glutamine, and 1X Antibiotic-Antimycotic (Thermofisher Scientific), in a humidified incubator at 37 °C and 5% CO_2_. The cells were frozen in culture media supplemented with 10% DMSO (Sigma-Aldrich) at a concentration of 5 × 10^6^ cells per ml and transferred to a cryogenic freezing container (Nalgene) in a −80 °C freezer for 24 h prior to long term storage in liquid nitrogen. To prepare the cells for cfDNA extraction, the cells were harvested by centrifugation at 5000 g for 5 min. DNA extraction was undertaken using the Qiagen QIAamp DNA Mini Kit (Hilden, Germany) according to the manufacturer’s protocol.

The concentrations of extracted DNA were determined using Nanodrop spectrophotometers (Thermo Fisher Scientific) by adding 1 µl of the untreated DNA to 9 µl of DNase-free water. The ratio of 260/280 nm and 260/230 nm optical density was examined and any sample that yielded values in the range 1.8–2.0 and 2.0 to 2.2 respectively was selected for use. To prepare the DNA sample for fragmentation, both human and bacterial samples were divided in a 5 ml Eppendorf tubes, making a total volume of 2 ml for each experiment. They were maintained on ice during fragmentation to avoid spontaneous bubbling and sample evaporation. Vibra Cell VCX-130 (Sonics, Newtown, CT) with a 2-mm probe was used for fragmentation. To this end, the key step was to establish a method to generate randomly distributed lengths of DNA fragments representative of circulating cfDNA. To achieve this, we used a sonication technique because (1) it does not carry-over chemicals such as enzymes that may affect the stress response, (2) it does not create artefacts by deleting raw sequence threads, and (3) it is relatively inexpensive and reproducible. The principle behind this approach is that high frequency sound waves when subjected to brief periods on the DNA sample is abruptly converted into mechanical force variations, which can cause DNA samples to fragment. To identify the critical fragment size, we investigated the interplay of incident power (*p*) with a fixed time by tuning the size distribution of the genomic DNA. To confirm the fragmentation of DNA using this technique, gel electrophoresis to separate DNA fragments based on their size and charge was performed (Fig. 7a). DNA samples from three species were analysed, (1) *Escherichia coli* as a model for Gram-negative bacteria, (2) *Staphylococcus aureus* for Gram-positive bacteria and (3) human as a control. As expected in the absence of sonication, no detectable fragments were observed. Additionally, when *p* ≥ 80% was performed, there was sample loss due to spontaneous bubbling and evaporation. Thus 0 < *p* < 80% was the range where the actual fragmentation was effective. For a genomic DNA with insufficient power (*p* < 10%), both bacteria and human DNA were not fragmented and produced a band with approximately the same mobility as untreated DNA. Remarkably, *p* = 10% is a finite power threshold where there is a transition of genomic DNA from a constrained ordered state to a randomly disordered state with a concomitant preference for smaller sized fragments. We ascertained the validity of this phenomenon (Fig. 7a), by comparing the fragment size distributions on the gel at the different power settings (10% ≤ *p* ≤ 60%). The analysis as shown in Fig. 7a reveals that the whole double DNA helix cannot ultimately withstand the build-up of mechanical forces beyond *p* ≥ 10% and so the genomic DNA becomes fragmented, resulting in different size distributions on the gel. The fragmentation was performed using 15 pulses where each pulse lasted for 15 s and switched for 30 s to cool at 40% incident power. The process was repeated for all the 15 pulses. Considering the incident power and the fragment size distribution, *p* = 40% was chosen for further experiments as this resulted in a consistent fragment with size distribution ≤ 300 bp regardless of the species of origin. Before use, the DNA fragments were denatured at 70 °C for 5 min and cooled on ice to enable denatured single-stranded segments of the DNA hybridization with the corresponding complementary surface construct.

### Surface constructs

The readout sequences are based on the oligonucleotides functionalized at the 5′ end with a thiol group via a hexyl spacer (Supplementary Table 1). The minimum length of 27 bases was determined to provide a unique sequence that is specific to each bacterial species, confirmed by qPCR (Supplementary Fig. 2). The sequences computationally predicted were custom made (Microsynth). Prior to use, the thiol-modified sequences were extracted with ethyl acetate (Sigma Aldrich) for five times to remove thiol-protecting Dithio Threitol (DTT). The purity of the resulting sequences was determined by Nanodrop spectrophotometers (Thermo Fisher Scientific) before use. The sensing sequences at 100 µM were mixed in equal parts with 100 µM triethyl ammonium acetate buffer (TEAA, Sigma-Aldrich), pH 7 and dissolved to yield a concentration of 50 µM. The micro-capillary glass tubes (King Precision Glass, Claremont, CA, USA) were arranged on a functionalization stage according to the pitch size of 250 μm filled with 50 µM solution of proxy sequences. The nanomechanical detection systems were functionalized on both sides in parallel and under identical conditions for 20 min and rinsed in 1 M NaCl sodium citrate (SCC) buffer solution (Sigma Aldrich). Unused chips were stored for several days at 4 °C without significant loss in performance.

### Measurements

The surface modified nanomechanical detection system was installed in the liquid chamber (80 µl), filled with 10% human blood serum plasma diluted in 1 M NaCl sodium citrate buffer (SCC), pH = 7.4. The experiments were performed using a time-multiplexed optical detection system at constant flow (rate, 30 µl min^−1^) and a pumping system (GENIE, Kent Scientific) to control the exchange of up to six different solutions. The mechanical deflection was measured by reflecting a laser beam off the free end. The deflection data was subsequently converted into a differential stress between the upper and lower sides of the detection system (see Supplementary Section ‘Conversion of the bending signals into differential stress’). These nanomechanical detection systems are susceptible to multiple factors such as changes to index of refraction, temperature, fluidic disturbances, non-specific interactions, and thermal drifts. They are cancelled by acquiring differential measurements which enable in situ comparison between signals from capture agents and plant-based agents, *Arabidopsis* species (see Supplementary Table 1) as negative controls. Both bacteria and human samples were suspended at specific concentrations in 10% human blood serum plasma diluted in 1 M NaCl sodium citrate buffer (SCC), pH = 7.4.

### Quantitative polymerase chain reaction (qPCR)

Whole genomic DNA extracted from human and bacteria was hybridized to TaqMan probe designed to mimic model bacteria (Supplementary Table 1). The amplification was quantified at a final concentration of 1 µl of forward primer (10 µM), 1 µl of reverse primer (10 µM), 0.5 µl of TaqMan probe (10 µM), 10 µl of 2X TaqMan Gene Expression Mix (Applied Biosciences, Foster City, CA), 2 µl of 100 ng, 10 ng or 1 ng extracted human and bacterial genomic DNA and 5.5 µL of DNase-free water (InvitrogenTM, Thermo Fisher Scientific). The qPCR was performed on the 7500 Fast Real-Time PCR System (Applied Biosystems) with hot-start activation (2 min at 50 ◦C, 10 min at 95 ◦C) and 40 reaction cycles (15 s at 95 ◦C, 30 s at 60 ◦C and 60 s at 72◦C to collect fluorescence. The experiments were performed at 100 ng, 10 ng and 1 ng with 100 ng human DNA as a control measurement to ensure selectivity and reproducibility.

### Data analysis

The statistical analysis was performed on each set of measurement and error bars represent the standard error of the mean of triplicate values of stress data from three separate chips, each with a total of 72 measurements. The statistical analysis was performed by using IBM SPSS 24 Statistics software (https://www.ibm.com/spss) and Origin 2016 (9.3) software (https://www.originlab.com/). The results are summarised in the Supplementary Fig. 1.

## Electronic supplementary material

Below is the link to the electronic supplementary material.

Supplementary Material 1

Supplementary Material 2

## Data Availability

All data generated or analysed during this study are included in this published article (and its supplementary information file).
