# Supplementary material for: Nanomechanical detection to empower robust monitoring of sepsis and microbial adaptive immune system-mediated proinflammatory disease
Source: Sci Rep. 2024 Dec 2;14:29979. doi: 10.1038/s41598-024-80126-6 (PMC11612153; doi:10.1038/s41598-024-80126-6)
Supplement: Supplementary file 2 — Supplementary Material 2 [file 41598_2024_80126_MOESM2_ESM.pdf]

Nanomechanical detection to empower robust monitoring of sepsis and microbial adaptive immune system-mediated proinflammatory disease

Kessarin Thanapirom1, Walid Al-Akkad, Aylin Pelut, Zahra Sadouki, Jemima B. Finkel, Stefan Nardi-Hiebl, Wieland Vogt, Benjamin Vojnar, Hinnerk Wulf, Timothy D McHugh, Krista Rombouts, Massimo Pinzani, Emmanouil Tsochatzis and Joseph W. Ndieyira. Ndieyira

Supplementary Table 1

Table 1 Sequences computationally predicted

Target	Read sequence	Use
Model bacteria	HS-(CH2)6-GTACAAGGCCCGGGAACGTATTCACCG	Detection of DNA (Figs. 2, 3, 4 & 5) and cDNA (Table 2)
Gram-negative bacterial species	HS-(CH2)6-GACGTAAGGGCCATGATGACTTGACGTC	Detection of DNA (Figs. 2, 3, 4 & 5) and cDNA (Table 2)
Gram-positive bacterial species	HS-(CH2)6-GACGTCAAATCATCATGCCCCTTATGTC	Detection of DNA (Figs. 2, 3, 4 & 5) and cDNA (Table 2)
TaqMan probe	6-FAM-TACAAGGCCCGGGAACGTATTCACCG-TAMRA	Amplification investigation using qPCR
Reference based on *Arabidopsis* species	HS-(CH2)6-TTTGACTTAGGGCTTCATCAC	For reference control measurement for DNA detection (Figs. 2, 3, 4 & 5)

Supplementary Table 2

Table S2 Solution targets titrated in actual blood serum carriers

Target	Target sequence
Model bacteria	CGGTGAATACGTTCCCGGGCCTTGTAC
Gram-negative bacterial species	GACGTCAAGTCATCATGGCCCTTACGTC
Gram-positive bacterial species	GACATAAGGGGCATGATGATTTGACGTC
forward primers	5'-AACTGGAGGAAGGTGGGGAT-3'
reverse primers	5'- AGGAGGTGATCCAACCGCA-3'

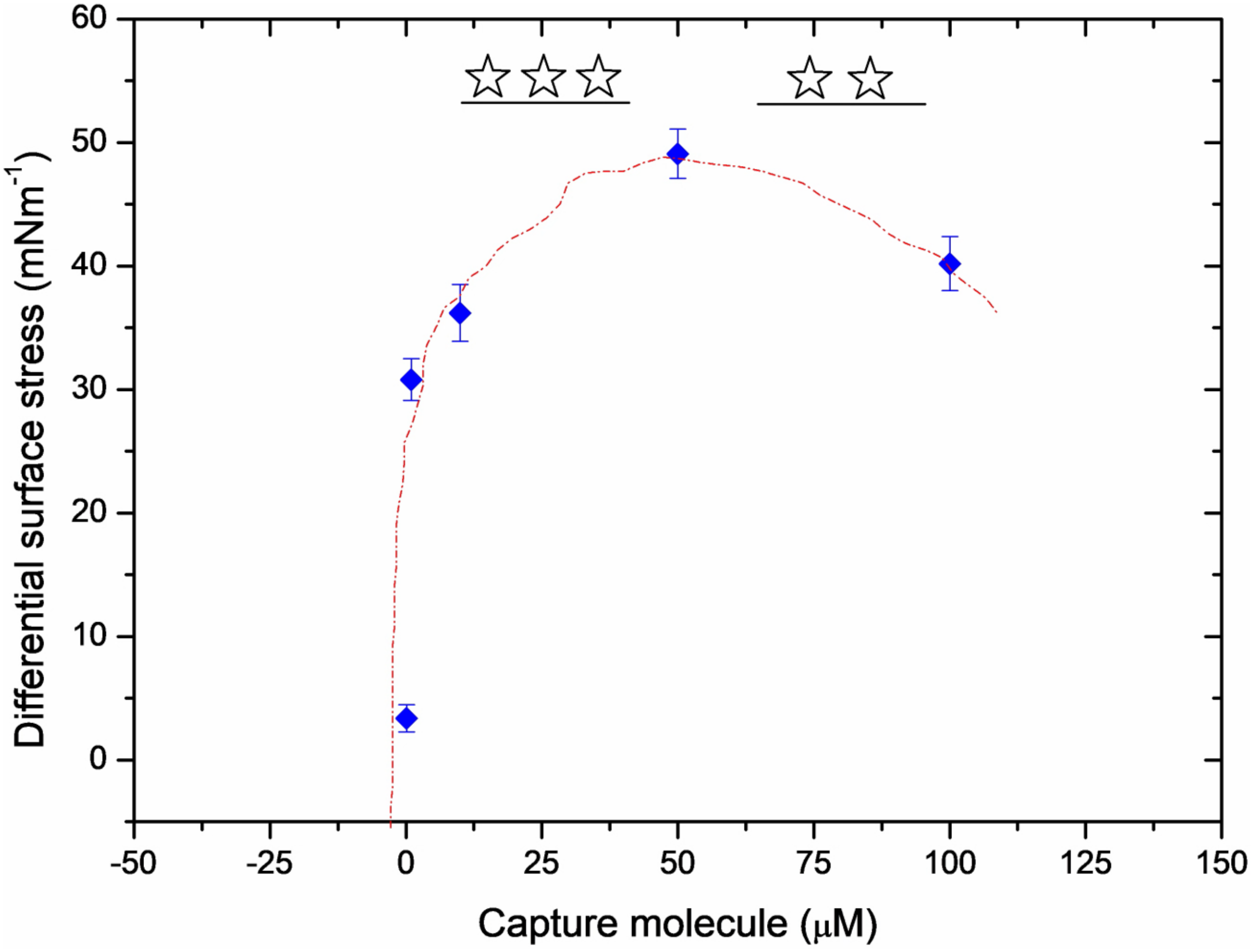

Supplementary Figure 1: **Optimization signal response**. Relative mechanical bending response relative to the receptor concentration in saline phosphate buffer solution. The dotted line is a guide for the eye. Statistical significance of mechanical response between the receptor concentration at 50 µM relative to that at 10 µM and 100 µM were determined using a One-way ANOVA followed by a Bonferroni's post-hoc test. ** $p<0.01$, and *** $p<0.001$. Each data point represents the mean of triplicate values. Error bars represent the standard error of the mean (SEM). Therefore, the receptor concentration at 50 µM was selected for the sensor surface construct using sequences computationally predicted.

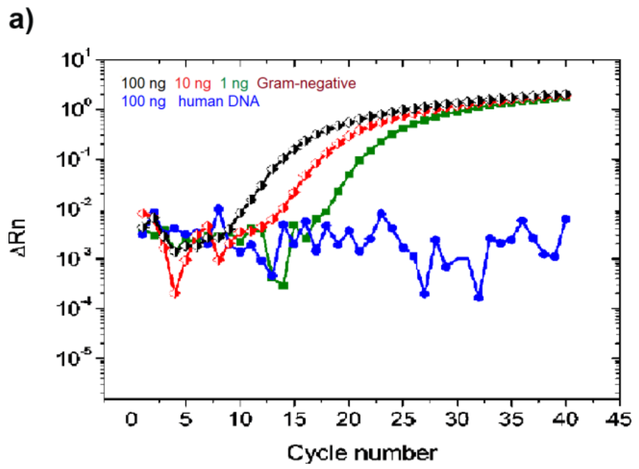

Supplementary Figure 2: **qPCR detection of whole genomic DNA extracts** (**a**) qPCR amplification plot of 100 ng (black line with solid symbols), 10 ng (red line with solid symbols) and 1 ng (green line with solid symbols) genomic DNA derived from Gram-negative bacteria in DNase-free water showing a growth fluorescent signal phase at around cycle number 10, 15 and 20, respectively. (**b**) qPCR amplification plot of 100 ng (black line with solid symbols), 10 ng (red line with solid symbols) and 1 ng (green line with solid symbols) genomic DNA derived from Gram-positive bacteria in DNase-free water showing a growth fluorescent signal phase at around cycle number 10, 15 and 20, respectively. In **a,b**, Human-derived genomic DNA in DNase-free water (blue line with solid symbols) show no change in fluorescent signal throughout the 40 cycles. We imposed a 40-cycle cut-off threshold because any readings beyond this cycle number was considered unreliable.

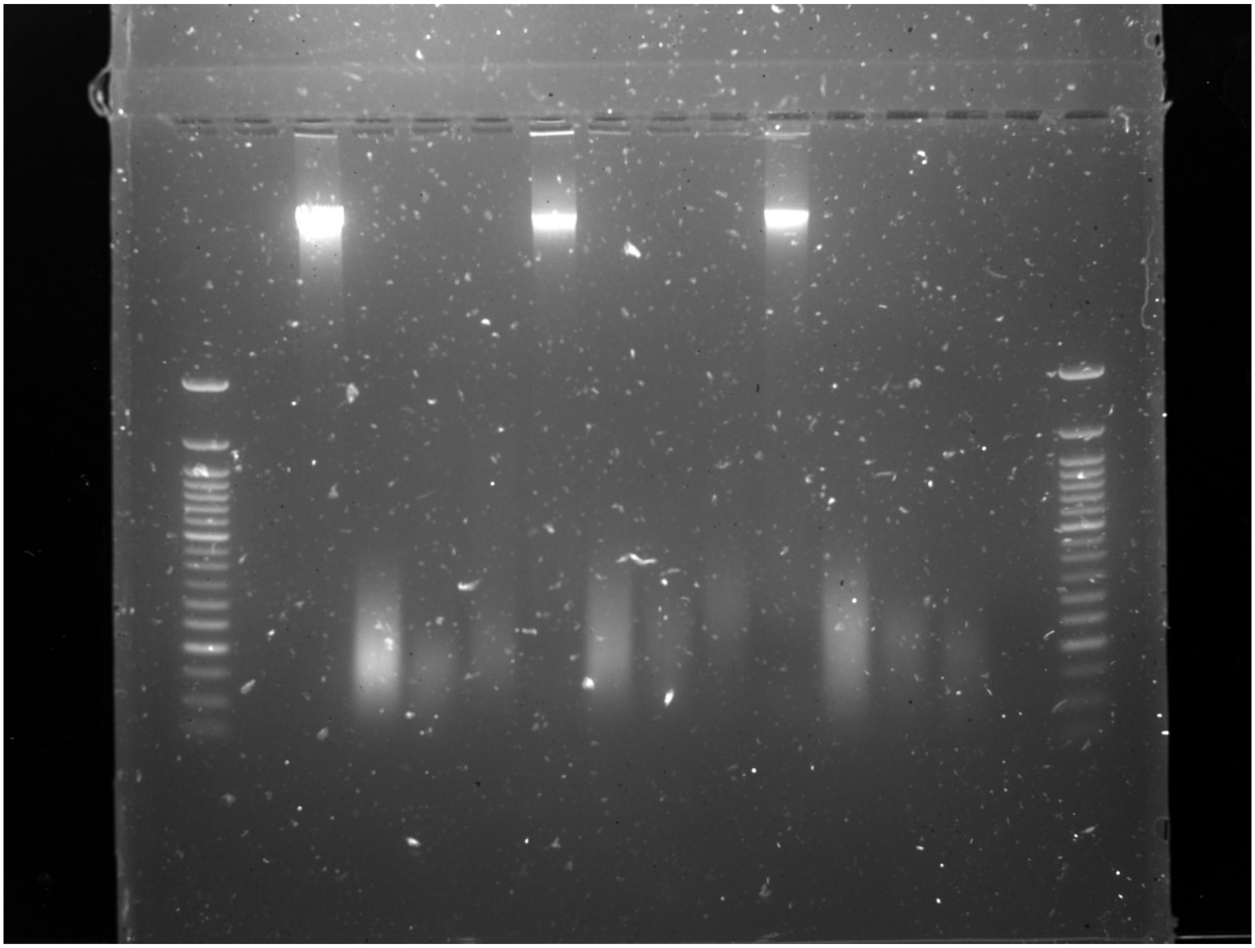

Supplementary Figure 3a: Full length gel membrane as shown in Fig. 7a of the main text.

METHODS & MATERIALS

Design and modification of bacterial sequences computationally predicted

To achieve effective quantitation of bacterial signatures in blood microbiome for differential diagnosis in various clinical settings, the readout sequences must (1) distinguish bacteria from other species in the blood (2) provide species-specific signature sequences useful for identification of bacteria and (3) capable of detecting the signature sequences with high reproducibility and sensitivity even in a complex biological background with other competing molecules such as human DNA and proteins. The nucleotide sequences were derived from 16S rRNA gene, a common standard for classification and identification of bacteria. We incorporated a six-unit carbon thiol group at the 5' to act both as a linker and spacer and to facilitate self-assembly on the surface of the nanomechanical sensing element (Supplementary Table 1). As a sample to verify the efficiency of hybridization test on the model bacteria before extending to viable bacteria, the nucleotide sequences were reverse transcribed to obtain cDNA (Supplementary Table 2). To enable acquiring of differential measurements to eliminate non-specific binding, we adopted the sequences based on the plant, Arabidopsis species (Supplementary Table 1) under the assumption that 16S rRNA genes is bacteria species-specific and does not allow cross-reactive reactions from plant species.

Preparation of cantilever nanomechanical sensing element

We used commercially available arrays of silicon cantilevers, 500 µm long, 100 µm wide and 1 µm thick with a spring constant of 0.02 N/m. The cantilever array sensors used in these experiments were custom-made and obtained from Micromotive GmbH, situated at Carl-Zeiss-Str. 18-20, 55129 Mainz, Germany, and from IBM Research GmbH, Switzerland. The cantilever

array sensors were first cleaned with freshly prepared piranha solution at a ratio 1:1 H_2SO_4 and H_2O_2 (Sigma Aldrich) for 20 mins as previously described[1]. The arrays were then thoroughly rinsed in deionized water before being immersed in the second freshly prepared piranha solution for another 20 mins, and again rinsed thoroughly with deionized water. Finally, the arrays were rinsed with pure ethanol and dried on a hotplate at 70 °C for 20 secs. They were then inspected using the optical microscope to confirm their cleanliness before transferring to the evaporation chamber (BOC Edwards Auto 500, U.K.) for overnight pumping. The upper side of each cantilever was coated using e-beam evaporation with a 2 nm titanium layer followed by 23 nm of gold, and at evaporation rates of 0.02 nm/s (titanium) and 0.07 nm/s (gold), as measured directly above the source by quartz crystal monitor.

Functionalization using glass cappilary

PEG, plant proteins and monoclonal antibodies

We first defined the percentage ratios of receptors (plant proteins, anti-TSPAN7, anti-CA19-9 and anti-albumin) by incorporating a surface linker molecule (HS-C11-(Eg)3-OCH2-COONHS) (ProChimia Surfaces, Poland) where Eg is the ethylene glycol group and NHS is the N-Hydroxysuccinimide group molecule with a second SAM-forming molecule PEG (HS-C11-(Eg)3-OMe) where Me is the methyl group. PEG and NHS thiol solutions were mixed at a ratio of 1:9 respectively to yield a total concentration of 50 µM in pure ethanol and was used to functionalize nanomechanical cantilever arrays. The cantilever arrays were first inserted into the micro-capillary glass tubes and the mixed ethanolic thiol solutions of 'inert' SAM–PEG and SAM–NHS were injected into the respective micro-capillary glass tubes. The arrays were incubated for 20 mins, rinsed with ethanol and dried in air followed by (5 mM, pH 5.4) sodium acetate buffer solution for

5 mins to activate the surface for the coupling reaction. The solutions (50 µg/ml in PBS buffer at pH 7.4) of plant proteins, anti-TSPAN7, anti-CA19-9 and anti-albumin antibodies were injected across cantilever arrays and incubated overnight at 4 ºC for coupling reactions to the activated NHS thiolated binding sites. After an overnight incubation, the microarrays were subjected to a "capping" procedure, using 1 M ethanolamine, pH 8.5 to de-activate unreacted NHS thiols and rinsed thoroughly in PBS buffer at pH 7.4 three times before use.

SAMs of inert PEG molecules and DNA probe molecules were diluted to a concentration of 50 µM in pure ethanol. The individual nanomechanical cantilevers were functionalized by incubating them with the individual SAMS of PEG and DNA probe with a cysteine thio-linker for 20 min. This was followed by a wash in pure ethanol, distilled water and PBS solution. Both sides of each cantilever array were functionalized with SAMS of PEG and DNA as described in the main text.

Conversion of the bending signals into differential surface stress

The absolute deflection at the free end of each cantilever, Δz_{abs}, was measured using a time-multiplexed optical detection system in different buffer and blood serum environments under constant flow. To convert the bending signals into differential surface stress, the raw data from each set of separate cantilever chips were analyzed off-line using automated data software. The bending signals were subsequently converted into differential surface stress between the upper and lower sides of the cantilever, $\Delta\sigma_{abs}$, according to the Stoney's equation[2]

$$\Delta\sigma_{abs} = \frac{1}{3}\left(\frac{t}{L}\right)^2 \frac{E}{1-\nu}\Delta z_{abs} \quad (1)$$

where L is the effective length of the cantilever up to 500 μm, t is the thickness (∼1 μm) and $E/(1 - \nu) = 180$ GPa is the ratio between the Young's modulus E and Poisson ratio ν of Si(100). The differential equilibrium surface-stress was calculated by subtracting in-situ reference stress signal from the measuring absolute stress signal. The positive bending response corresponds to downward bending of the cantilevers due to compressive surface stress and upward bending of cantilevers is caused by tensile surface stress.

REFERENCES

(1) Ndieyira, J., Watari, M., Barrera, A. *et al*. Nanomechanical detection of antibiotic–mucopeptide binding in a model for superbug drug resistance. *Nature Nanotech* **3,** 691–696 (2008).

(2) Stoney, G. G. The tension of metallic films deposited by electrolysis. *Proc. R. Soc. Lond. A* **82**, 172–175 (1909).
